# A systematic review of technology-infused physical activity interventions in K-12 school settings: effectiveness, roles, and implementation strategies

**DOI:** 10.1186/s12966-025-01811-x

**Published:** 2025-08-23

**Authors:** Taemin Ha, Jongho Moon, Hyeonho Yu, Xiaoping Fan, Lisa Paulson

**Affiliations:** 1 Department of Family, Nutrition, and Exercise Sciences, City University of New York–Queens College, New York, USA; 2 Department of Kinesiology, Montclair State University, New Jersey, USA; 3 Department of Educational Studies, Ball State University, Indiana, USA; 4 Department of Physical Education, State University of New York at Cortland, New York, USA; 5 Department of Applied Human Sciences, University of Minnesota Duluth, Minnesota, USA

**Keywords:** Public health, Health promotion, School health, Whole-school, Exercise, Physical education, Digital competence, Child, Adolescent

## Abstract

**Background:**

Rapid technological advancements have rendered many prior reviews of technology-integrated physical activity (PA) interventions in K–12 schools obsolete. A comprehensive analysis examining both the effects of these interventions and the specific roles that technology plays has been notably lacking. This review aimed to systematically examine the effects of technology-infused PA interventions and identify the specific types, roles, and contextual applications of technology within K–12 schools.

**Methods:**

This systematic review adhered to the Preferred Reporting Items for Systematic reviews and Meta-Analyses (PRISMA) guidelines. A thorough search across seven electronic databases (CINAHL, ERIC/EBSCOhost, PsycINFO, PubMed/MEDLINE, Scopus, SPORTDiscus, and Web of Science) included studies published up to May 20, 2024. Only randomized controlled trials (RCTs), cluster-RCTs, or rigorous quasi-experimental designs with matched/statistically controlled comparisons (N-RCTs) examining technology-infused PA interventions for school-aged children and adolescents were considered.

**Results:**

Fifty-eight studies met inclusion criteria. Wearable devices (e.g., accelerometers) were the most used for PA measurement. Other technologies like web-based platforms, mobile applications, and exergaming served as educational tools, communication platforms, or core intervention components. Interventions occurred primarily in classrooms, followed by physical education spaces, and extended to home/online environments, school breaks (e.g., recess), and before-school time. Overall, interventions positively affected PA levels and related outcomes (e.g., PA enjoyment), though effectiveness varied by technology type, design, and context.

**Conclusions:**

Technology holds substantial potential to enhance PA promotion in schools, but its effectiveness hinges on well-designed interventions that consider the specific types and applications of technology.

**Supplementary Information:**

The online version contains supplementary material available at 10.1186/s12966-025-01811-x.

## Background

Regular engagement in physical activity (PA) confers significant physical, mental, social, and emotional health benefits, playing a critical role in the prevention of non-communicable diseases [1, 2]. Despite these well-documented benefits, recent data indicate a global shortfall in PA among youth. Over 80% of adolescents aged 11–17 years worldwide do not meet the recommended levels of PA, contributing to rising rates of obesity and other chronic health conditions [3]. In the United States, only 20–28% of those aged 6–17 years achieve the daily recommendation of 60 min of PA averaged across the week, including muscle-strengthening activities [4], and these figures mirror broader global trends. If these trends persist, international healthcare systems may face cumulative costs of approximately INT$ 524 billion (US$300 billion) over the next decade in treating diseases that could be prevented through increased PA [5]. These alarming statistics highlight the urgent call for action needed for effective PA interventions that can reverse this trend.

Schools, where children and adolescents spend a significant portion of their day, offer an ideal environment for integrating PA into daily routines. Their infrastructure provides numerous opportunities for movement through physical education classes, active classrooms, recess, and extracurricular activities. Leveraging this existing infrastructure, multicomponent approaches to promote coordinated PA have emerged and been promoted and implemented internationally in recent years. These frameworks—such as the Active School Culture [6] and the Comprehensive School Physical Activity Program (CSPAP) model [7] in the United States, as well as the Creating Active Schools (CAS) model [8] from the United Kingdom and Finland’s Schools on the Move program [9]—emphasize the importance of integrating PA opportunities across the entire school day and engaging all stakeholders. Within this conceptual context, technology has emerged as a powerful tool in educational settings, enhancing PA opportunities by incorporating innovative solutions (e.g., instruction, tracking, engagement, and communication). To ensure success, it is essential to systematically evaluate these technology-infused PA interventions to understand their impact and scalability in real-world educational settings. The promising conceptual research thus far coupled with the lack of school-based implementation clearly necessitates additional interventions and review—a gap this article aims to cover.

Over the past few decades, technology has become an integral part of both educational and health promotion strategies [10, 11]. Over 70% of teachers now use technology in their classrooms, and innovative tools—ranging from interactive mobile applications (apps) and active video games to extended reality and virtual exercise programs—have been shown to improve academic achievement and may also enhance PA levels [12–17]. Specifically, technology-integrated PA programs provide students with interactive, engaging experiences that not only motivate them to move more but also allow them to track their progress and participate in group activities [18, 19]. Further, these tools hold promise for increasing student engagement in PA by providing interactive, personalized feedback and fostering social connectivity [20]. Despite the growing potential of these technological innovations, the literature has yet to thoroughly investigate the nuanced roles that technology plays in enhancing PA within diverse school-based contexts. This gap in the literature, particularly regarding the timing, context, and specific functions of technology within PA interventions, represents an important area for investigation.

Although previous systematic reviews have examined the efficacy of technology-based PA interventions for youth [18, 21, 22] or focused on specific technology tools such as virtual and augmented reality [23], these studies are relatively outdated due to the rapid advancements in technology. Moreover, no study has simultaneously investigated both the effects and roles of technology in PA interventions within K–12 school environments. Specifically, the distinct types and roles of technology, as well as the timing and contextual factors of these interventions within the school day, remain underexplored. To address this gap, the purpose of this review was to systematically examine the effects of technology-infused PA interventions, specifically in K–12 school settings, and to identify the distinct roles that technology plays in these interventions. “Technology-infused” refers to the active role technology plays during any stage of an intervention period, from preparation or training to assessment or evaluation. This review was guided by the following five research questions:

1. What types of technology are used in PA interventions in K–12 school settings?
2. What roles does technology serve in PA interventions in K–12 school settings?
3. During which contexts of the school day (e.g., physical education classes, recess, after-school programs) are technology-infused PA interventions implemented?
4. To what extent do technology-infused interventions improve PA–related outcomes (e.g., participation rates, enjoyment, fitness, or motivation) in K–12 school settings?
5. What is the quality of the evidence reported in the research?

By addressing these questions, this review contributes to a deeper understanding of how technology can improve PA promotion in K–12 schools, identifies best practices for incorporating technology into PA programs, and informs future policy and intervention development.

## Methods

### Protocol and registration

This review was conducted in accordance with the Preferred Reporting Items for Systematic reviews and Meta-Analyses (PRISMA) 2020 guidelines (see Supplementary Document, Appendix A) [24], and the protocol was registered with the International Prospective Register of Systematic Reviews (PROSPERO; Registration number: CRD42024538424).

### Search strategy and terms

A search was conducted across seven electronic databases between May 15–20, 2024, including CINAHL, ERIC (EBSCOhost), PsycINFO, PubMed/MEDLINE, Scopus, SPORTDiscus, and Web of Science. No restrictions were applied regarding the date or country of publication. Guided by the Population, Intervention, Comparison, Outcomes, and Study (PICOS) framework [25], the search strategy incorporated key elements such as the study population (e.g., elementary and K-12), intervention and outcomes (e.g., PA and technology), and study design (e.g., randomized controlled trials [RCTs] and quasi-experimental designs). The decision not to specifically limit the control or comparison group’s intervention approach during the search stemmed from the recognition of the diverse nature of interventions in the existing literature. Interventions often vary significantly in their approach, intensity, duration, and other characteristics; thus, a broader search strategy was employed to capture a comprehensive range of studies. Key terms used in the PICOS framework are detailed in Table 1.

**Table 1:** Search strategy and terms

Database	Population	Intervention and Outcome		Study Design
		Physical Activity	Technology	
CINAHL; ERIC; PsycINFO; SCOPUS; SPORTDiscus; Web of Science	K-12 OR P-12 OR School OR “Primary school” OR “Secondary school” OR Elementary OR “Middle school” OR “High school” OR Student OR Child* OR Adolescen* OR Youth OR Teen* OR Famil*	“Physical activity” OR Exercise OR “Physical education”	Technology OR Digital	Intervention OR Experiment OR Program* OR Evaluation OR Trial OR Random* OR Clinic* OR "Controlled trial"
PubMed/MEDLINE	K-12[tiab] OR P-12[tiab] OR School[tiab] OR “Primary school”[tiab] OR “Secondary school”[tiab] OR Elementary[tiab] OR “Middle school”[tiab] OR “High school”[tiab] OR Student[tiab] OR Child*[tiab] OR Adolescen*[tiab] OR Youth[tiab] OR Teen*[tiab] OR Famil*	“Physical activity”[tiab] OR Exercise[tiab] OR “Physical education”[tiab]	Technology[tiab] OR Digital[tiab]	Intervention[tiab] OR Experiment[tiab] OR Program*[tiab] OR Evaluation[tiab] OR Trial[tiab] OR Random*[tiab] OR Clinic*[tiab] OR "Controlled trial"[tiab]

* The asterisk indicates a truncated search term used to capture variations of a word (e.g., 'adolescence,' 'adolescent,' 'adolescents')

### Eligibility criteria

This review included studies involving participants aged 6–19 years, encompassing both children and adolescents. Eligible interventions had to incorporate technology in any capacity as part of PA programs or initiatives within K–12 school settings. The technology could serve various functions, such as a measurement tool, communication tool, instructional or training tool, or feedback device. For an intervention to be considered “technology-infused,” the technology was required to play an intentional role during the intervention period. This meant either directly engaging participants—for example, through feedback, instructional content, or gamified experiences—or serving as a core component of intervention implementation or evaluation, such as the systematic use of accelerometers to assess PA-related outcomes and psychological factors. Interventions conducted within any school context, including physical education classes, active classrooms, or before- and after-school programs, were also eligible for inclusion. Only studies employing RCTs, cluster-RCTs (C-RCTs), or rigorous quasi-experimental designs with matched or statistically controlled comparisons (N-RCTs) were considered. Furthermore, studies were required to be published in English, with no restrictions on publication date or geographical location.

The review focused exclusively on typically developing children and adolescents aged 6–19 years. Studies were excluded if they involved participants outside this age range or focused on students with clinically diagnosed physical, intellectual, or developmental disabilities (e.g., Attention-Deficit/Hyperactivity Disorder [ADHD]) or conditions such as cerebral palsy or traumatic brain injury/concussion. Further exclusions applied to studies that did not explicitly use technology during or for the intervention or lacked a focus on PA-related outcomes. For example, studies that inferred technology use (e.g., presumed use of computers or internet for communication) but did not explicitly describe its integration were not included. Additionally, studies reported solely as abstracts, theses/dissertations, conference proceedings, or unpublished literature were excluded from this review.

### Study selection

All search records from the seven selected electronic databases were imported into Covidence, a web-based platform designed to streamline systematic review processes (https://www.covidence.org). Duplicate records were automatically identified and removed. In the first screening stage, two reviewers independently assessed each title and abstract for inclusion or exclusion based on the eligibility criteria. Any conflicts between the two reviewers were resolved by the principal investigator. In the second stage, two reviewers independently screened the full texts of studies that passed the initial screening. Each study was assessed for inclusion or exclusion, and any conflicts arising during this stage were also resolved by the principal investigator. All reviewers were thoroughly briefed on the study’s purpose and had a clear understanding of the eligibility criteria, ensuring a consistent and systematic approach throughout the screening process.

### Data extraction and synthesis

For each included study, two reviewers independently extracted the relevant data. Any discrepancies or conflicts were resolved by the principal investigator. The data extraction process followed the systematic review framework and aligned with the specific objectives of this review. The extracted data included study characteristics, such as the study’s purpose, design, sample characteristics, findings, and other relevant details. Additionally, data on the technology used in the interventions were collected, including the types and roles of technology, as well as the specific contexts in which the technology-infused PA interventions took place.

Data extracted from the studies were organized in Microsoft Excel, with each piece of information pertaining to school characteristics and technology categorized individually. The data were then summarized, tabulated, and compared. Quantitative measures of PA (e.g., *p*-value, effect size, percentage increase/decrease) were extracted and narratively synthesized alongside study characteristics and key findings. This approach avoided implying a formal statistical analysis beyond descriptive presentation. Given the considerable heterogeneity in study design, intervention characteristics, outcome measures, and reporting formats, a formal meta-analysis was not conducted. Instead, findings were summarized descriptively to present trends in effectiveness and contextual factors across the included studies.

### Quality assessment

Two reviewers independently assessed the quality of each included study, with the principal investigator resolving discrepancies and making final decisions. The Cochrane Risk of Bias version 2 tool (RoB 2) was employed to evaluate the risk of bias in RCTs and C-RCTs [26]. RoB 2 encompasses six domains and provides an overall judgment. The five domains are: (a) bias arising from the randomization process; (b) bias arising from the timing of identification and recruitment of individual participants in relation to the timing of randomization; (c) bias due to deviations from the intended interventions; (d) bias due to missing outcome data; (e) bias in the measurement of outcomes; and (f) bias in the selection of the reported result. Based on responses (e.g., yes, probably yes, probably no, no, not applicable, no information) to a series of signaling questions outlined in the guidance document, judgments for each domain were categorized as “low risk of bias,” “some concerns,” or “high risk of bias.”

For N-RCTs, the Risk of Bias in Non-randomized Studies of Interventions tool (ROBINS-I) was used [27]. This tool assesses seven domains and provides an overall judgment. The seven domains are: (a) bias due to confounding; (b) bias in the selection of participants; (c) bias in the classification of interventions; (d) bias due to deviations from the intended intervention; (e) bias due to missing data; (f) bias in the measurement of outcomes; and (g) bias in the selection of the reported result. As recommended by the PRISMA guidelines, a domain-specific risk of bias assessment was conducted to determine the overall risk of bias for each study; these items were not summed numerically; instead, each criterion was evaluated individually [24].

## Results

**Fig. 1:**
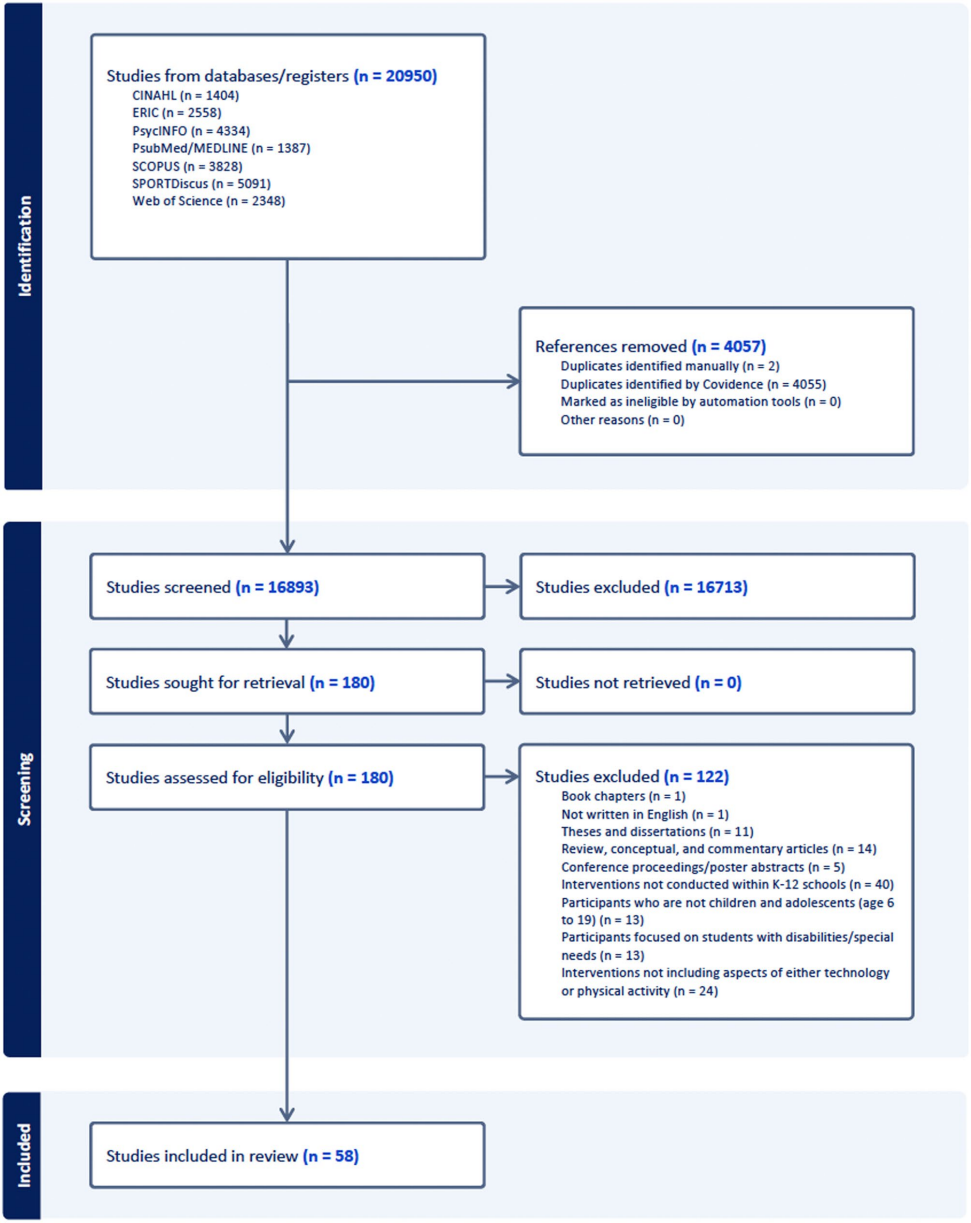
Study identification process using PRISMA framework

Searches across seven databases identified a total of 20,950 records, with 4,057 duplicates removed. After screening the titles and abstracts of 16,893 articles, 180 were selected for full-text assessment. Ultimately, 58 studies met the inclusion criteria and were included in the analysis. The study selection process is illustrated in Fig. 1.

### Study characteristics

Across the 58 included studies, a total of 15,236 participants were reported. Of these, 10,839 participants were in intervention groups, and 4,424 were in control groups. Notably, 21 studies did not include a control group. Additionally, two studies reported only the total number of participants, without specifying the breakdown by group, despite having a control group. One study did not report participant numbers at all but provided data for 16 schools. Sample sizes ranged from a minimum of 4 participants (reported in two studies) to a maximum of 1,914. Participant ages in intervention groups primarily spanned from 6 to 19 years; however, one study included participants aged 5–10, and another included ages 15–20. Some studies lacked clear reporting on participant gender or age.

Of the included studies, 22 were conducted in North America (e.g., United States of America, Mexico), 15 in Europe (e.g., United Kingdom, Netherlands), 11 in Oceania (e.g., Australia, New Zealand), six in Asia (e.g., Hong Kong, China, Singapore), and two in the Middle East (e.g., Turkey, United Arab Emirates), while two studies were conducted in multinational countries—one involving eight countries and one involving five countries. While 43 studies did not report urbanicity, only 15 studies did. Among these, 13 studies were conducted in urban settings, while two studies took place in rural settings. In terms of research design, 19 studies employed an RCT design, while 39 studies used an N-RCT design. The earliest publication year among the 58 studies was 2004, and the most recent publication was in 2024. Table S1 in the Supplementary Document presents details of the characteristics of the selected studies, including information about the purpose statement, study design, country of origin, sample size, and a description of the intervention (program).

### Types of technology in the interventions

**Fig. 2:**
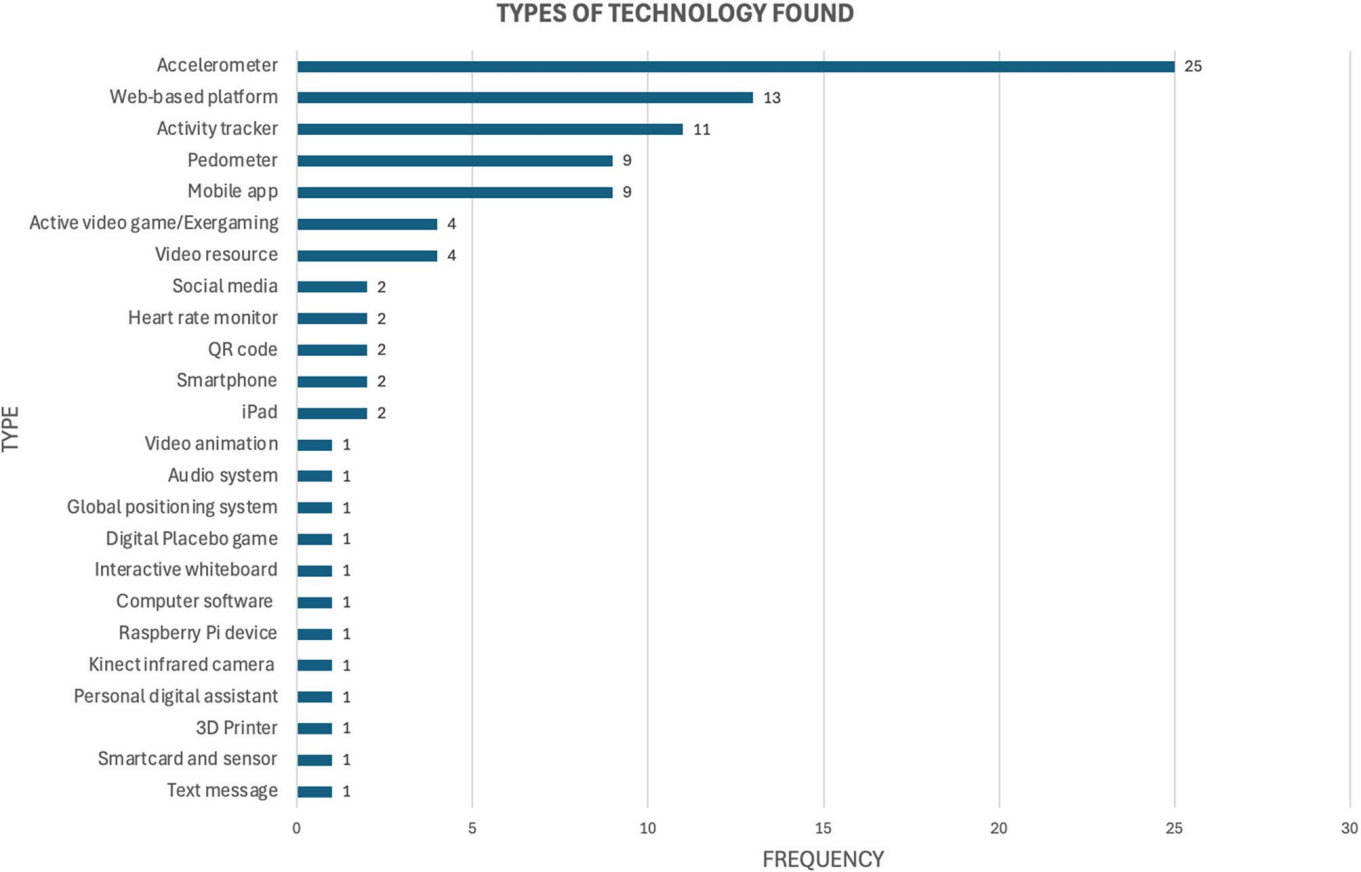
Types of technology found in PA interventions

In the 58 technology-infused PA intervention studies, a total of 96 different technologies were clearly identified or mentioned. The most frequently used technology was the accelerometer, which appeared in 25 studies, while the least frequently used technologies appeared just once across 12 different categories (e.g., 3D printer, interactive whiteboard, global positioning system). Some studies explicitly mentioned specific wearable devices, such as accelerometers (*n* = 25) or pedometers (*n* = 9). However, 11 technologies were simply referred to as “activity trackers,” with or without brand or product names, including wristbands or any type of smartwatch. Figure 2 illustrates the frequency of technology types found in the interventions.

### Roles of technology in the interventions

**Fig. 3:**
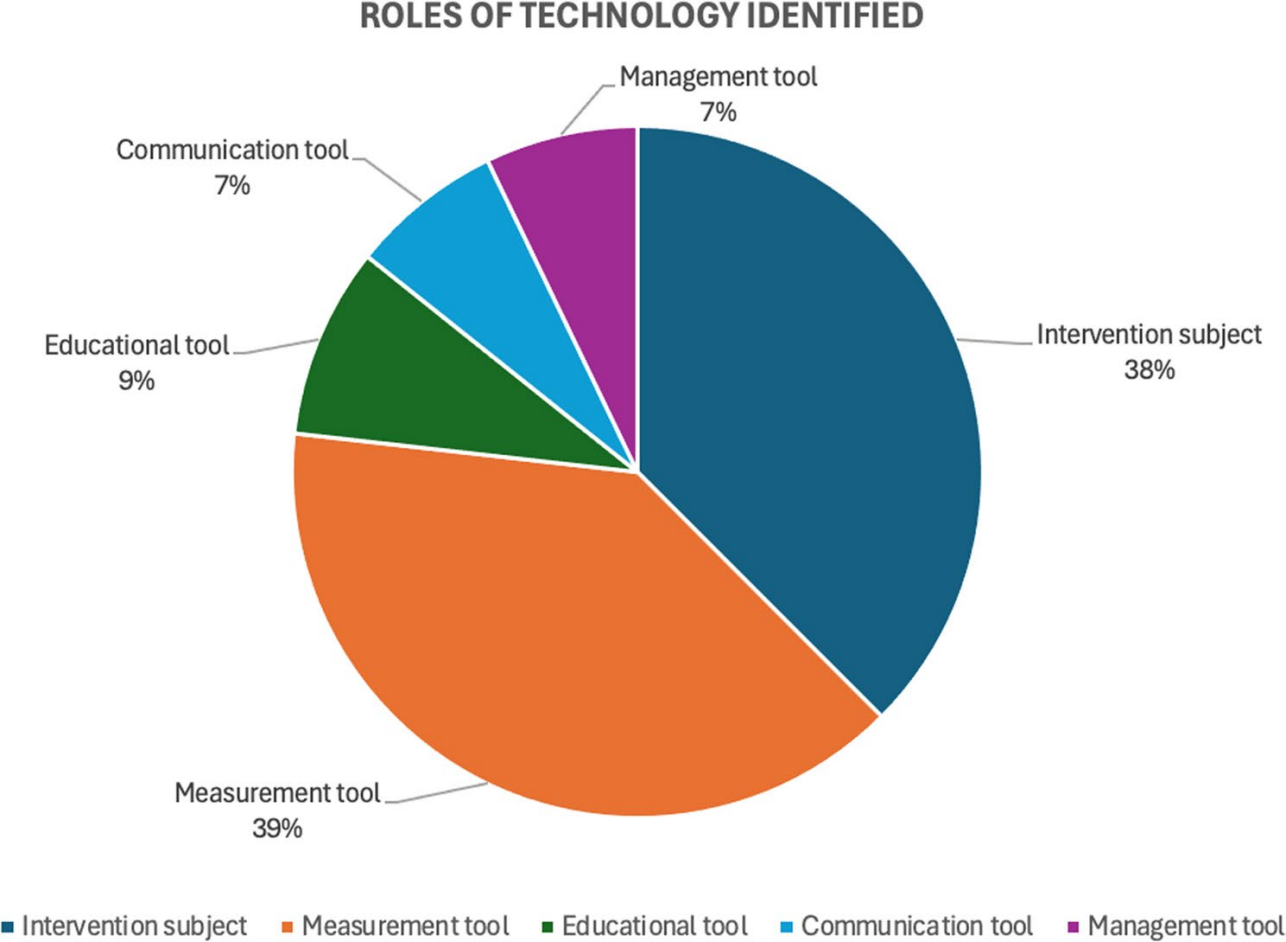
Roles of technology identified in PA interventions

In terms of the roles these technologies played in the interventions, 112 roles were identified across the studies and categorized into five areas: measurement tool (*n* = 44; 39%), intervention subject (*n* = 42; 38%), educational tool (*n* = 10; 9%), communication tool (*n* = 8; 7%), and management tool (*n* = 8; 7%). For the “intervention subject” category, certain technologies were the central focus of the intervention, such as exergaming. Technologies used as “measurement tools” included accelerometers and pedometers, which were frequently employed to track PA levels. “Educational tools” encompassed technologies like web-based platforms and video resources, which were used to educate educators, students, and other personnel involved in the interventions. “Communication tools” facilitated interactions between teachers and students, teachers and parents, or other stakeholders in the intervention, often using social media or smartphones. Finally, technologies used as “management tools” included web-based platforms or mobile apps, which helped organize and manage the intervention. Figure 3 shows the distribution of roles of technology identified in the PA interventions.

### Contexts of the interventions

**Fig. 4:**
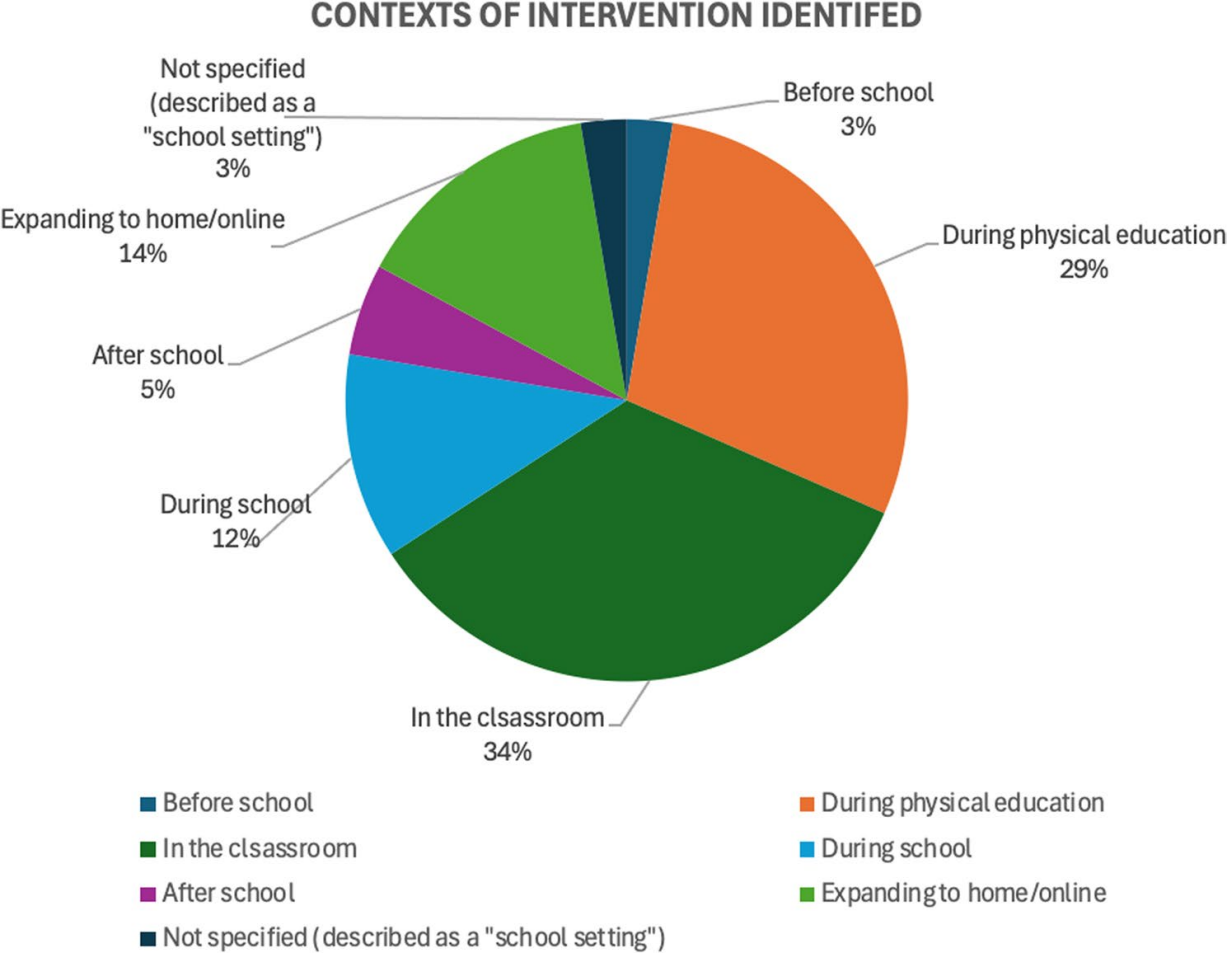
Contexts of technology-infused PA interventions identified

In terms of the contexts of technology-infused PA interventions,76 contexts were identified across the 58 studies, which were grouped into six categories. Two studies did not clearly specify the context but only mentioned a “school setting.” The six contexts include: in the classroom (*n* = 26; 34%), during physical education (*n* = 22; 29%), expanding to home or online (*n* = 11; 14%), during school (*n* = 9; 12%), after school (*n* = 4; 5%), and before school (*n* = 2; 3%). The “during school” context includes recess, lunchtime, or breaks. “Expanding to home/online” refers to interventions that extend beyond the school setting—such as during physical education—allowing participants to continue PA-related activities at home or online. Among the 58 studies, 18 studies (including the two studies that only mentioned “school setting”) implemented interventions across multiple components (e.g., interventions during both physical education and after-school programs), while 40 studies focused on a single context. Figure 4 illustrates the distribution of these identified contexts for technology-infused PA interventions. Table S2 in the Supplementary Document presents details of technology-related information from the selected studies, including the types and roles of technology in the interventions and the contexts of the interventions.

### Effectiveness of technology-infused PA interventions

In terms of PA-related outcomes, across the 58 studies, 48 measured PA levels, including eight studies that assessed not only PA levels but also other PA-related outcomes. However, 10 studies did not measure PA levels but focused on other PA-related variables, such as attitudes toward PA, PA knowledge, PA enjoyment, motivation toward PA in leisure time, and PA behavior, including time spent playing outside. Among the studies measuring PA levels, 41 used wearable technology (e.g., accelerometers and Fitbits) as a data collection instrument, while seven used self-reported questionnaires, a systematic observational tool (e.g., System for Observing Student Movement in Academic Routines and Transitions [SOSMART]), or an activity log system. Among the 48 studies that measured PA levels, 35 (73%) reported a statistically significant increase in PA outcomes. Additionally, of the 10 studies that focused on other PA-related outcomes (e.g., PA enjoyment, motivation), 9 (90%) showed statistically significant improvements. In total, 44 of the 58 included studies (76%) reported statistically significant findings for at least one PA-related outcome. Table S3 in the Supplementary Document presents details on the effectiveness of technology-infused PA interventions, including information on PA-related variables, statistical results, and key findings from the studies.

### Risk of bias and study quality

Overall, reviewer agreement for the quality assessment exceeded 80% for both the RoB 2 [26] and ROBINS-I [27] tools. The quality assessment results showed that among the 19 RCT studies, 10 were considered low risk, while nine were deemed to have some concerns. For the 39 N-RCT studies, eight were considered low risk, and 31 were considered moderate risk. These results, from both RoB 2 and ROBINS-I, are presented in Figure S1 and Figure S2 in the Supplementary Document.

## Discussion

This systematic review aimed to examine the effects and roles of technology in PA interventions within K–12 school settings. A total of 58 studies were included in the analysis, providing insights into the various types of technology used, their roles in the interventions, the contexts in which these technologies were implemented, and the effectiveness of these interventions in improving PA outcomes. The findings highlight several key themes related to the role of technology in enhancing PA and suggest important directions for future research and practice.

### Types and roles of technology in PA Interventions

The current review identified wearable devices, such as accelerometers, activity trackers, and pedometers, as the most commonly utilized technology in PA interventions. These devices primarily served as measurement tools, providing feedback and effectively motivating students to engage in PA. The high prevalence of accelerometers, found in 24 of the 58 studies, likely reflects their relative affordability, ease of use, and objectivity in measuring PA, a point also supported by Ferguson et al.‘s review [28]. However, an over-reliance on accelerometers as sole measurement tools might represent a missed opportunity to leverage their full potential for motivational feedback and behavior change, as suggested by Wang et al. [29]. These findings align with prior research and further extend understanding by suggesting that future studies should explore how wearable devices can be leveraged not only for tracking but also for providing continuous motivational support to enhance PA engagement among students within school contexts [30].

These findings also reveal a relatively lower prevalence of technology used for direct PA promotion compared to its use for assessment (e.g., monitoring, data collection), which may stem from several factors. Firstly, assessment technologies like accelerometers and activity trackers are well-established, widely accessible, and relatively easy to implement, potentially explaining their dominance. In contrast, designing and delivering engaging, developmentally appropriate, and scalable technology-based strategies for PA promotion (e.g., exergaming, interactive mobile applications) often requires substantial technological infrastructure, curricular integration, and pedagogical innovation. Moreover, a substantial portion of the current literature appears to focus on validating the feasibility and accuracy of technological tools rather than examining their sustained behavioral impact. This trend highlights a research gap and underscores the need for future studies to prioritize the development and evaluation of technology-driven interventions that not only measure but actively promote PA in school settings. Investigating how these tools can be embedded within multicomponent, school-wide strategies may yield more comprehensive and impactful outcomes. These potential factors warrant further exploration in future research.

Web-based platforms and mobile apps—serving not only as intervention subjects but also as educational tools—were the next most commonly used technologies in the 58 studies (22 studies, including 13 web-based platforms and nine mobile apps). These technologies are often used for their practicality and versatility; for instance, step challenge websites were used both to assess their effectiveness in promoting PA and to educate students, teachers, and staff about the importance of PA and its integration into daily routines [31, 32]. Furthermore, these platforms and apps were often used in conjunction with wearable devices to track, monitor, and analyze PA data [33–35]. This integration of multiple technologies suggests that a combined, synergistic approach may enhance intervention effectiveness by offering a more holistic means of tracking and encouraging PA—a promising avenue that warrants further investigation.

In addition to serving as measurement or educational tools, several technologies also functioned as the central focus of certain interventions, accounting for 38% of the identified technology roles. Active video games, or exergaming, are examples of technologies serving as the central focus of interventions. Exergaming represents an emerging concept in the integration of technology into PA interventions [36, 37]. This highlights technology’s potential to act as an engaging and motivating activity in itself—actively improving PA levels and related outcomes rather than merely serving as a monitoring tool. This shift represents a crucial change in using technology as an agent of change, given its ability to be self-sustaining and reduce the burden on teachers who may feel overwhelmed by integrating new PA opportunities, as noted by Webster et al. [38]. For example, several studies have demonstrated that video resources used for classroom brain breaks provide additional opportunities to integrate various technologies into classrooms, further enhancing PA promotion [39–41]. Future research should rigorously evaluate the long-term effectiveness and scalability of exergaming and other interactive, technology-infused PA interventions. Specifically, researchers should explore strategies for their sustainable integration into school curricula, incorporating recommended best practices to ensure effective implementation while maximizing student engagement and PA levels.

Additionally, technologies were identified as serving both communication and management roles in the interventions, though these were the least frequently identified roles, each accounting for only 7% of the total roles. As communication tools, technologies such as mobile apps and social media facilitated interactions between teachers, students, and parents, fostering a sense of community and support around the initiatives [31, 42, 43]. Literature suggests that these communication channels are valuable for both school staff and students to discuss health and PA-related topics within their communities [44]. Regarding management tools, technology played a crucial role in organizing and tracking the progress of interventions. For example, learning management systems were used to manage student performance data throughout the intervention [45, 46]. The underutilization of technology in these capacities highlights a significant gap and presents a promising opportunity for future development. Effective communication and streamlined intervention management through technology could enhance implementation fidelity, ensuring that interventions are carried out as intended while also improving scalability, making it easier to expand these interventions across various school settings [47, 48]. Further research into the role of technology as a communication or management tool could lead to more efficient and effective PA interventions, ultimately benefiting school communities by promoting long-term PA engagement and fostering sustained improvements in student health outcomes.

### Contextual considerations

Technology-infused PA interventions were implemented across a variety of school contexts in the studies included in this review. The most common settings were physical classrooms (both general and physical education), followed by home or online contexts, during-school periods (e.g., recess, lunchtime, or breaks), after-school programs, and before-school time. Interestingly, although physical education is typically the primary time for students to engage in PA during the school day [6, 7, 49], the classroom emerged as the most frequently identified context for PA interventions in this review. These findings suggest that classrooms provide a viable and accessible venue for integrating PA, with technology playing a significant role in facilitating classroom-based activities. This is particularly important in light of the growing pressure on schools to maximize academic learning time. Integrating PA into the classroom through technology could address both physical and academic goals simultaneously. However, to ensure technology-driven PA activities augment versus compromise academic priorities, teachers must possess adequate knowledge of PA principles and receive appropriate structural support [38, 50]. When successfully integrated, technology can maximize its benefits, as noted by Ha et al. [51]. Future research should focus on finding the optimal balance between PA integration and academic instruction in classroom settings, ensuring that both physical and academic development are supported. Additionally, research should investigate the effectiveness of technology integration in this context.

The during-school context also presents valuable opportunities for students to engage in PA. These unstructured periods, such as recess, lunchtime, or breaks, are ideal for promoting spontaneous, self-directed activity [6, 7, 49], and technology can play a significant role in facilitating this engagement [18, 52, 53]. Additionally, interventions extending beyond the school day—incorporating activities at home or online—highlight the importance of a holistic, multi-context approach to PA promotion [54]. Extending interventions outside the traditional school setting could be a critical component of a comprehensive school-based PA strategy, as it reinforces PA-related behaviors and actively involves families in supporting PA outside of school hours [55, 56]. By incorporating technology across multiple contexts—such as the classroom, physical education, before- and after-school programs, and even home settings—PA interventions can reinforce PA-related messages and behaviors, leading to more sustained changes in students’ activity levels. Future research should explore a multi-tiered, technology-infused PA intervention approach, ensuring that interventions are adaptable across different contexts to maximize both their effectiveness and sustainability. This approach could offer a comprehensive solution to addressing the growing need for PA promotion in school communities and contribute to long-term improvements in student health outcomes.

### Effectiveness of technology-infused PA interventions

Drawing from the included studies, technology-infused PA interventions generally demonstrated positive effects on PA levels. However, the effectiveness varied depending on the type of technology used, its application, and the context in which the intervention was implemented. Interventions where technology was the central focus—such as exergaming or video resources—were generally effective [36, 37, 39, 40], as were those involving wearable devices [57–59]. Nevertheless, some studies reported no significant increases in PA levels or related outcomes, possibly due to insufficient statistical power to detect effects, or because the chosen outcome measures were not sensitive enough to capture changes in PA behavior [34, 35, 60, 61]. These results should be interpreted with caution, as the variability likely stems from differences in intervention duration, intensity, context, and the diversity of technologies used. The mixed outcomes underscore the need for standardizing intervention protocols and carefully considering contextual factors, such as intervention duration, intensity, and the specific role of the technology, in future research. Standardization would improve the comparability of findings and provide clearer insights into the most effective technology-based strategies for promoting PA in school settings.

### Quality of evidence

The included studies varied in quality. Of the 19 RCTs, 10 studies considered a low risk of bias, while 9 had some concerns. The remaining 39 studies were N-RCTs, with eight studies considered low risk and 31 studies considered moderate risk. This heterogeneity limits the strength of causal inferences that can be drawn, and studies with a higher risk of bias may have overestimated positive effects [26, 27]. The variability in study quality underscores the need for more high-quality, well-designed trials to better understand the long-term impact of technology-infused PA interventions in school settings.

### Limitations of the review and future research

This review had several limitations. Firstly, many included studies lacked detailed information on key variables such as participant gender and age, and several did not include control groups, potentially affecting the generalizability of the findings. The impact of interventions can vary significantly across these variables, particularly different age groups. While the design of this systematic review and the heterogeneity of the included studies precluded a robust subgroup analysis by age to definitively identify such effect modification, this remains a critical area for further investigation. Future studies should consider stratified or age-targeted analyses to better understand how developmental stage influences the effectiveness and implementation of technology-infused PA interventions. Secondly, feasibility and acceptability metrics (e.g., implementation fidelity, user satisfaction, teacher or student burden, and sustainability) were infrequently reported in the included studies. These indicators are essential for translating interventions from research to practice and should be systematically included in future investigations. Thirdly, the diversity of technologies and intervention approaches across studies made it challenging to draw definitive conclusions about the most effective strategies or methods. Finally, limiting the search to English-language publications may have excluded relevant studies published in other languages.

## Conclusions

This systematic review highlights the significant potential of technology-infused PA interventions to increase PA levels and improve PA-related outcomes among K–12 students within school settings. However, the effectiveness of these interventions is highly dependent on the specific technology used, its role within the intervention, and the quality of its implementation context. Given that today’s students are highly exposed to various technological tools, technology should be considered essential—not optional—for effectively facilitating PA within school environments and beyond. By incorporating a diverse array of technological tools and adopting a multi-context approach, future interventions can more effectively engage students and foster healthier, more active school communities.

Ongoing research and innovation are critical for translating these findings into effective practices and policy changes. Future studies should prioritize conducting rigorous RCTs to compare various technologies and intervention strategies. The variability in study quality underscores the need for well-designed, high-quality trials to assess the long-term effects of technology-infused PA interventions. Longitudinal studies are essential to evaluate sustained impacts on PA, sedentary behavior, and overall health outcomes. Furthermore, qualitative research can provide valuable insights into the facilitators and barriers to the successful implementation and sustainability of these technology-driven PA programs.

## Supplementary Information

Supplementary Material 1.

## Data Availability

No datasets were generated or analysed during the current study.

## Abbreviations

PA: Physical activity
CSPAP: Comprehensive School Physical Activity Program
CAS: Creating Active Schools
PRISMA: Preferred Reporting Items for Systematic reviews and Meta-Analyses
PICOS: Population, Intervention, Comparison, Outcomes, and Study
RCT: Randomized controlled trial
N-RCT: Non- randomized controlled trial
RoB 2: Cochrane Risk of Bias version 2
ROBINS-I: Risk of Bias in Non-randomized Studies of Interventions
