# Supplementary material for: A systematic review of technology-infused physical activity interventions in K-12 school settings: effectiveness, roles, and implementation strategies
Source: Int J Behav Nutr Phys Act. 2025 Aug 23;22:113. doi: 10.1186/s12966-025-01811-x (PMC12374309; doi:10.1186/s12966-025-01811-x)
Supplement: Supplementary file 1 — Supplementary Material 1. [file 12966_2025_1811_MOESM1_ESM.docx]

**A Systematic Review of Technology-Infused Physical Activity Interventions in K-12 School Settings: Effectiveness, Roles, and Implementation Strategies**

**Supplementary Document**

Taemin Ha^1*^, Jongho Moon^2^, Hyeonho Yu^3^, Xiaoping Fan^4^, and Lisa Paulson^5^

^1^Department of Family, Nutrition, and Exercise Sciences, City University of New York–Queens College, USA

^2^Department of Kinesiology, Montclair State University, USA

^3^Department of Educational Studies, Ball State University, USA

^4^Department of Physical Education, State University of New York at Cortland, USA

^5^Department of Applied Human Sciences, University of Minnesota Duluth, USA

* Corresponding Author:

Taemin Ha, Ph.D.

Assistant Professor,

Department of Family, Nutrition, and Exercise Sciences

City University of New York–Queens College

65-30 Kissena Blvd. Flushing, NY 11367, USA

taemin.ha@qc.cuny.edu

**Table S1**

*Study characteristics*

| **Source** | **Purpose Statement** | **Study Design** | **Country (Urbanicity, if available)** | **Sample size (*n*) and Participant Characteristics** | | **Intervention (program) Description** |
| --- | --- | --- | --- | --- | --- | --- |
|  |  |  |  | **INT** | **CONT** |  |
| Balasekaran et al., 2021 [1] | To examine the effects of classroom-based Brain Breaks^®^ PA Solution on the attitudes of Southeast Asian Singaporean primary school students towards PA. | N-RCT: Quasi-experimental design | Singapore  Urbanacity:  Not reported | *n* = 48  Age: 8-11 (9.71±0.99)  Gender: 66 females and 47 males  Demographic: Not reported | *n* = 65  Age: 8-11  Gender: 40 females and 25 males  Demographic: Not reported | Title: Brain Breaks^®^ PA Solution  Duration: 3-5 minute Brain Breaks^®^ videos during class time; 5 days per week; 10 weeks  Description: The primary goal of the Brain Breaks^®^ intervention is to improve students’ attitudes towards PA by making it a fun and integral part of their daily routine. The intervention leverages technology to promote PA in a way that is both effective and appealing to students. The Brain Breaks^®^ intervention consists of short video exercises, each lasting between three to five minutes. These videos are designed to be integrated into classroom settings, allowing students to engage in PA during academic lessons. |
| Botagariyev et al., 2024 [2] | To examine the effectiveness of digital and web-based technologies in middle school PE, including impacts on PA, academic performance, and motivation. | N-RCT: Quasi-experimental design | Kazakhstan  Urbanacity: Urban | *n* = 338  Age: 12-14 (13.2±1.05)  Gender: 191 females and 147 males  Demographic: Not reported | N/A | Title: Digital Technology-Based PE Training Course  Duration: Full academic semester (Sep–Dec 2022), 3 PE classes per week, 50 total lessons.  Description: Students used accelerometers to track PA during PE classes. A closed Facebook group was used for teacher-student communication, sharing activity data, and class-related discussions. |
| Brackney et al., 2021 [3] | To examine if a before-school, play-based dance program could help second-graders meet PA guidelines and assess its feasibility. | N-RCT: Quasi-experimental design | United States  Urbanacity: Urban | *n* = 11  Age: 7-8  Gender: Not reported  Demographic: Not reported | *n* = 10  Age: 7-8  Gender: Not reported  Demographic: Not reported | Title: Play-Based Dance Program  Duration: 8 weeks, 3 days/week  Description: Before-school dance sessions in a multi-purpose room. Participants wore activity trackers to measure PA levels. The control group followed their regular school routine and wore trackers for comparison |
| Buchele Harris & Chen, 2018 [4] | To examine the effects of a 4-week technology-enhanced PA intervention on students’ daily PA and aerobic fitness. | N-RCT: Quasi-experimental design | United States  Urbanacity: Not reported | *n* = 60  Age: 10-11  Gender: Not reported  Demographics: 60% identified as non-white (30% African American) | *n* = 56  Age: 10-11  Gender: not reported  Demographics: 48% identified as non-white (19% African American) | Title: Technology-Enhanced PA Intervention  Duration: 4 weeks, 5 days/week  Description: Physical Activity Engaging the Brain with Fitbit Challenge (PAEB-C) group did daily PA with Fitbit challenges, Fitbit-O group only wore Fitbits. The control group followed their regular school schedule. |
| Caillaud et al., 2022 [5] | To evaluate the effectiveness of the iEngage digital health education program in increasing PA among adolescents. | N-RCT: Quasi-experimental design | Australia  Urbanacity: Urban | *n* = 57  Age: 10-12  Gender: Not reported  Demographics: not reported | n = 26  Age: 10-12  Gender: Not reported  Demographics: not reported | Title: iEngage  Duration: 5 weeks  Description: A digital health education program with 10 interactive learning modules integrating PA tracker data to help students set goals, self-monitor, and assess progress. The control group did not receive the iEngage program. |
| Chen et al., 2020 [6] | To assess the efficacy of a HIIT-based fitness education unit in middle school PE. | N-RCT: Quasi-experimental design | United States  Urbancity: Urban | *n* = 113  Age: Middle school (grades 6-8)  Gender: Not reported  Demographics: Not reported | *n* = 119  Age: Not reported  Gender: Not reported  Demographics: Not reported | Title: HIIT-Based Fitness Education  Duration: 8 weeks, 2-3 times per week  Description: HIIT-focused fitness education designed to improve PA knowledge and health-related fitness. The control group continued the traditional multi-activity PE curriculum. |
| Coknaz et al., 2019 [7] | To examine the impact of active video games on PA, reaction time, self-perception, and enjoyment levels in inactive children. | RCT | Turkey  Urbanacity: Urban | *n* = 53  Age: 8.2-13.1  Gender: Not reported  Demographics: Not reported | N/A | Title: Active Video Games  Duration: 12 weeks, 50-60 min, 3 days/week  Description: Children engaged in active video games targeting whole-body movement. The control group did not participate in the active video game intervention. |
| Cook et al., 2014 [8] | To evaluate the effectiveness of the web-based Activ-O-Meter in promoting PA among adolescents. | RCT | Multinational: Austria, Greece, Belgium, Germany, and Sweden  Urbanacity: Not reported | *n* = 536  Age: *M*_age_ = 14.38  Gender: 51% male  Demographics: Not reported | N/A | Title: Activ-O-Meter  Duration: 3 months  Description: An internet-based intervention providing tailored PA feedback. Participants received reports on PA levels, self-efficacy, and barriers. The control group was waitlisted. |
| Coombes & Jones, 2016 [9] | To assess the impact of “Beat the Street” on children’s active travel behavior. | N-RCT: Quasi-experimental design (Pilot) | United Kingdom  Urbanacity:  Urban | *n* = 51  Age: 8-10  Gender: 62.7% female  Demographics: Not reported | *n* = 29  Age: 8-10  Gender: 41.4% female  Demographics: Not reported | Title: Beat the Street  Duration: 9 weeks (20-week measurement period)  Description: Used tracking technology and rewards to encourage walking and cycling. The control group did not participate. |
| Corepal et al., 2019 [10] | To evaluate recruitment and retention for “StepSmart Challenge” and its impact on adolescent PA. | RCT | United Kingdom  Urbanacity: Urban | *N* = 224 (only total number of participants reported; includes three intervention schools and two control schools)  Age: 12-14  Gender: Not reported  Demographics: not reported | | Title: StepSmart Challenge  Duration: 22 weeks (Phase 1: 8 weeks, Phase 2: 14 weeks)  Description: Fitbit Zip-based PA competitions with material and social incentives. Control group did not receive the intervention. |
| Costigan et al., 2018 [11] | To examine the effects of HIIT on adolescent PA levels. | RCT | Australia  Urbanacity: Not reported | *n* = 43  Age: *M*_age_ = 15.8±0.6  Gender: Not reported  Demographics: Not reported | *n* = 22  Age: Not reported  Gender: Not reported  Demographics: Not reported | Title: Aerobic & Resistance HIIT Program  Duration: 8 weeks, 3 times/week  Description: The intervention group performed cardiorespiratory exercises, and the RAP group added resistance training. The control group continued usual PE and lunchtime activities. |
| Crossley et al., 2019 [12] | To assess the impact of personalized 3D-printed feedback on youth PA awareness, goal-setting, and motivation. | N-RCT: Quasi-experimental design | United Kingdom  Urbanacity: Not reported | *n* = *97*  Age: *M*_age_ = 7.9±0.3 for primary; *M*_age_ = 13.8±0.3 for secondary  Gender: Not reported  Demographics: Not reported | N/A | Title: 3D-Printing PA Intervention  Duration: 7 weeks with feedback sessions  Description: Participants received personalized 3D-printed models detailing their PA levels at multiple time points to enhance engagement and motivation. |
| Duck et al., 2021 [13] | To investigate the feasibility of using wearable activity tracker technology with altruistic motivation to increase PA and prosocial behavior in youth. | N-RCT: Quasi-experimental design | United States  Urbanicity: Rural | *n* = 17  Age: 9  Gender: Not reported  Demographics: Predominantly black | *n* = 18  Age: 9  Gender: Not reported  Demographics: Predominantly black (black children = 31) | Title: UNICEF Power Up Curriculum  Duration: 10 weeks  Description: Used Kid Power Bands to track PA, empowering children with points to support global nutrition efforts, coupling tech-use with charitable motives. |
| Duncan et al., 2019 [14] | To examine the effects of a compulsory, health-related homework programme on PA, dietary patterns, and body size in primary school-aged children. | RCT | New Zealand  Urbanacity: Urban | *n* = 346  Age: 7-10 (*M*_age_ = 8.71±0.987)  Gender: 171 males and 175 females  Demographics:  European 228 (65.9%), Maori 36 (10.4%), Pacific Island 22 (6.4%), Asian 46 (13.3%), and other 14 (4.0%) | *n* = 329  Age: 7-10 (*M*_age_ = 8.74±1.04)  Gender: 155 males and 174 females  Demographics:  European 232 (70.5%), Maori 18 (5.5%), Pacific Island 6 (1.8%), Asian 63 (19.1%), and other 10 (3.0%) | Title: A compulsory homework programme  Duration: 8 weeks  Description: The intervention involved an 8-week applied homework and in-class teaching module designed to increase PA and improve dietary patterns among primary school-aged children. |
| Erwin et al., 2013 [15] | To examine PA levels and intensity during indoor recess using PA dance videos. | N-RCT: Quasi-experimental design | United States  Urbanacity: Rural | *n* = 54  Age: 8-12  Gender: 27 females and 27 males  Demographic: 87% White, 4% African American, 4% Asian, 2% Hispanic, and 2% other. | N/A | Title: Children’s PA Levels During Indoor Recess Dance Videos  Duration: Not reported  Description: The intervention consisted of children participating in PA dance videos during indoor recess. |
| Evans et al., 2017 [16] | To examine the feasibility, acceptability, and preliminary effectiveness of using wearable PA monitors to increase PA in school-age children. | N-RCT: Quasi-experimental design | United States  Urbanacity: Urban | *n* = 32  Age: *M*_age_ = 12.3  Gender: 59% females in Phase 1, 31-60% females in Phase 2  Demographics: 45% White | *n* = 10  Age: *M*_age_ = 12.3  Gender: 60% females  Demographics: 45% White | Title: Not reported  Duration: 10 weeks (2 phases)  Description: In Phase 1 (4 weeks), participants wore a Fitbit Zip daily, aiming for 11,000 steps per day, and met weekly with study staff to sync data. In Phase 2 (6 weeks), they were assigned to one of three groups: (1) Fitbit Charge only, (2) Fitbit Charge with goal-setting and incentives, including weekly 40-minute sessions and rewards, or (3) a control group with no intervention. The study evaluated the impact of wearable trackers and behavioral interventions on PA levels. |
| Fogel et al., 2010 [17] | To examine the effects of exergaming on PA among inactive children in a PE classroom and to determine whether exergaming can increase PA levels and provide more opportunities for PA compared to standard PE programs. | N-RCT: Quasi-experimental design | United States  Urbanacity: Not reported | *n* = 4  Age: 9  Gender: 2 females and 2 males  Demographics: Not reported | N/A | Title: Exergaming-Based PA Program  Duration: Not specified  Description: The intervention involved the use of exergaming activities, such as Dance Dance Revolution (DDR) and 3 Kick, aimed at increasing PA levels among inactive students. Participants engaged with nine stations featuring 11 different activities using seven pieces of exergaming equipment. The program was designed to integrate interactive video gaming with physical movement to promote higher levels of engagement in PA. |
| Galy et al., 2019 [18] | To assess the reception and effectiveness of a technology-based educational program in encouraging Pacific adolescents to align their PA behaviors with international PA recommendations. | N-RCT: Quasi-experimental design | New Caledonia  Urbanacity: Not reported | *n* = 24  Age: 12-14  Gender: Not reported  Demographics: Not reported | N/A | Title: Not reported  Duration: iEngage program consists of eight self-paced modules, each including 21 minutes of learning activities and PA sessions.  Description: The iEngage program is a digital education app designed to enhance health literacy and physical skills among adolescents. Delivered through the BePatient platform, it includes eight self-paced modules featuring health-related content, goal setting, self-assessment tasks, quizzes, and short 2-5 minute PA sessions (totaling 21 minutes per participant). A Misfit Shine 2 activity tracker provided feedback to support self-monitoring of PA goals. |
| González-Cutre et al., 2014 [19] | To test the effectiveness of a Trans-Contextual Model-based intervention in promoting autonomy and healthy PA habits among students. The study examined whether the intervention increased perceived autonomy support from teachers, peers, and parents, enhanced self-determined motivation and related psychological factors, and ultimately led to higher PA levels during leisure time. | N-RCT: Quasi-experimental design | Spain  Urbanacity: Not reported | *n* = 21  Age: *M*_age_ = 11.28  Gender: 10 females and 11 males  Demographics: 100% White | *n* = 26  Age: *M*_age_ = 11.28  Gender: 13 females and 13 males  Demographics: 100% White | Title: Trans-Contextual Model-Based PA Intervention  Duration: Not reported  Description: The intervention was designed to promote autonomy and motivation for PA through PE lessons, including: Watching videos related to motivation and autonomy in adolescent PA; Engaging in discussions about the video content; Completing group tasks based on the videos; Conducting family discussions to extend the learning beyond the classroom. |
| Ha et al., 2022 [20] | To examine the criterion validity of two scalable, information technology-based systems designed to measure the quantity and quality of movement behaviors of children from Hong Kong primary schools. | Cross sectional study | Hong Kong  Urbanacity: Urban | *n* = 1204 (Validation sample for PA: 30 children; Validation sample for FMS Rater: 1174 children)  Age: 8.55±1.25 years for PA; *M*_age_ = 9.15 ± 1.63 for FMS Rater)  Gender: 52% male  Demographics: Not reported | N/A | Title: Fun to Move@JC Project  Duration: Not reported  Description: The Fun to Move@JC Project is a school-based, multi-component initiative launched in 2017, designed to enhance PA and physical literacy among primary school-aged children and their parents in Hong Kong. The project utilizes information technology to support physical education and children's engagement in PA. It includes the use of the Sport Band to measure PA levels and a system to assess fundamental movement skills. The program aims to promote healthy lifestyles and sustainable PA habits within the family context. |
| Hands et al., 2011 [21] | To examine the feasibility of a child-centred 24-week PA intervention, specifically the Play5 strategy, which encourages children to choose to play vigorously five times a day for about as long as school recess. | RCT | Australia  Urbanacity: Not reported | *n* = 132  Age: 7  Gender: 59 females and 73 males  Demographic: Not reported | *n* = 171  Age: 7  Gender: 80 females and 91 male  Demographic: Not reported | Title: Play5 Strategy  Duration: 24 weeks  Description: The Play5 strategy aimed to encourage children to engage in vigorous play five times a day, with each session lasting approximately as long as a typical school recess. The intervention included a 90-minute introductory session for each class, resources provided to teachers, parents, and children to support the implementation of the strategy, and parental involvement to encourage and support children’s PA. |
| Hartwig et al., 2019 [22] | To develop a PA monitoring system to track and provide feedback on MVPA during PE lessons, and validate its accuracy and reliability. | N-RCT: Quasi-experimental design | Australia  Urbanacity: Not reported | *n* = 592  Age: 492 secondary level and 100 elemetanry level (3-4 grades)  Gender: Not reported  Demographics: Not reported | N/A | Title: Custom PA Monitoring System  Duration: Not reported  Description: The study developed and tested a custom PA monitoring system using wireless tri-axial pedometers and a mobile app to provide feedback on MVPA during PE lessons. The system included pedometers that measured step counts to develop equations estimating %MVPA, and a mobile app that integrated with the pedometers to offer real-time MVPA feedback. To validate the system’s accuracy and reliability, ActiGraph accelerometers were used as a criterion measure. |
| Huang et al., 2019 [23] | To examine the effectiveness of a theory-based, technology-integrated website in promoting PA among schoolchildren. | N-RCT: Quasi-experimental design | Taiwan  Urbanacity: Not reported | *n* = 234  Age: *M*_age_ = 11.38 ± 0.34  Gender: 113 female and 121 male  Demographics: Not reported | *n* = 290  Age: *M*_age_ = 11.38 ± 0.34  Gender: 141 females and 149  Demographics: Not reported. | Title: Theory-Based, Technology-Integrated PA Promotion Website  Duration: Not reported  Description: The intervention utilized a technology-integrated website designed to promote PA among schoolchildren. Participants were divided into two intervention groups. The Self-Management Group had access to website tools that facilitated self-monitoring, goal setting, and incorporated storytelling elements based on a classical Chinese novel. The website also featured a geographical information system (GIS) mapping function to enhance engagement. The Animated Story-Only (Knowledge-Only) Group received a knowledge-based intervention through animated stories, but without access to the self-monitoring tools. |
| Jung et al., 2021 [24] | To describe the procedure for the development of a school-based PA program using an integrated approach through the modified intervention mapping protocol. | RCT | United States  Urbanacity: Not reported | *n* = 90  Age: 4^th^-5^th^ grade  Gender: Not reported  Demographics: Minority and socioeconomically disadvantaged students | *n* = 77  Age: 4^th^-5^th^ grade  Gender: Not reported  Demographics: Minority and socioeconomically disadvantaged students | Title: Project SMART  Duration: Not specified  Description: Project SMART is an online educational game where students navigate a virtual journey across the United States, propelled by their collective PA.  As students progress, they unlock modules aligned with STEM (Science, Technology, Engineering, and Mathematics) and social-emotional learning standards. The program aims to increase PA among 4th and 5th graders in minority and socioeconomically disadvantaged schools, enhancing self-determination for PA, academic engagement, and performance. The development of Project SMART involved applying Self-Determination Theory and a modified intervention mapping protocol, utilizing an integrative community participatory approach |
| Koorts et al., 2020 [25] | To examine the implementation of a wearable technology intervention (RAW-PA) to increase PA among inactive adolescents, evaluating perceptions of program acceptability, feasibility, perceived impact, and adolescent engagement and adherence. | RCT | Australia  Urbanacity: Not reported | *n* = 144  Age: *M*_age_ = 13.7±0.4  Gender: 51% male  Demographics: Not reported | *n* = =131  Age: *M*_age_ = 13.7±0.4  Gender: 49% male  Demographics: Not reported | Title: Raising Awareness of Physical Activity (RAW-PA)  Duration: 12 weeks  Description: The RAW-PA intervention aimed to increase MVPA among inactive adolescents through a multi-component approach. The intervention designed to be engaging and supportive, leveraging technology and social platforms to promote sustained increases in PA among adolescents in socioeconomically disadvantaged settings |
| Layne et al., 2022 [26] | To examine the effectiveness of models-based instruction and technology, specifically the use of heart rate monitors combined with the Sport Education Model, on MVPA and jump rope performance. | RCT | United States  Urbanacity: Not reported | *n* = 130  Age: 4^th^ grade  Gender: 61 females and 69 males  Demographics: Not reported. | N/A | Title: Comparison of Instructional Models and Technology Use in Physical Education  Duration: Not reported  Description: The study investigated the impact of different instructional models and the integration of heart rate monitors (HRMs) on students’ MVPA and jump rope performance. The integration of HRMs aimed to enhance students' awareness of their PA levels, while the Sport Education Model sought to create a more engaging and structured learning environment. |
| Lee et al., 2012 [27] | To examine the effect of an intervention combining self-efficacy theory and pedometers on promoting PA among adolescents. | RCT | Taiwan  Urbanacity: Not reported | *n* = 46  Age: *M*_age_ = 16  Gender: 100% female  Demographics: Not reported | *n* = 48  Age: *M*_age_ = 16  Gender: 100% female  Demographics: Not reported | Title: School-Based Physical Activity Intervention  Duration: 12 weeks  Description: The intervention was designed based on self-efficacy theory and incorporated the use of pedometers to promote PA among adolescent girls. |
| Lee & Gao, 2020 [28] | To examine the short-term (two-week) effects of mobile app-integrated physical education classes on children’s PA and psychosocial beliefs. | N-RCT: Quasi-experimental design | United States  Urbanacity: Not reported | *n* = 77  Age: 4^th^-5^th^ grade  Gender: Not reported  Demographics: Not reported | *n* = 80  Age: 4^th^-5^th^ grade  Gender: Not reported  Demographics: Not reported | Title: Mobile App-Integrated Physical Education Program  Duration: 2 weeks  Description: The intervention integrated iPads and mobile applications into PE classes to facilitate instruction and class management. The apps used included Educreations, an interactive whiteboard app for creating and sharing video lessons; GarageBand, a music creation app for designing rhythmic activities; Timer, an app for managing time during activities; and Team Shake, an app for randomly assigning students to groups or teams. Over the course of the two-week intervention, teachers used these apps to enhance lesson delivery and engage students in various PAs. The comparison group participated in traditional PE classes without the use of technology. |
| Lubans et al., 2014 [29] | To examine the development and implementation of a smartphone application designed to promote PA and reduce screen-time in adolescent boys considered “at-risk” of obesity. | RCT | Australia  Urbanacity: Not reported | *n* = 361 (only total number of participants reported)  Age: 12-14 years  Gender: 100% male  Demographics: Not reported | | Title: Active Teen Leaders Avoiding Screen-time (ATLAS)  Duration: 20 weeks  Description: The ATLAS program is a multi-component school-based intervention designed to prevent unhealthy weight gain by increasing PA, reducing screen-time, and lowering sugar-sweetened beverage consumption among adolescent boys attending schools in low-income areas. The intervention was guided by self-determination theory and social cognitive theory and included the following components: Teacher Professional Development, Provision of Fitness Equipment, Face-to-Face PA Sessions, Lunchtime Student Mentoring Sessions, Researcher-Led Seminars, Smartphone Application and Website, Parental Strategies |
| Lubans et al., 2018 [30] | To examine the effect of a school-based PA intervention on adolescents’ performance in mathematics and to explore potential mechanisms explaining the intervention’s effect on mathematics performance. | RCT | Australia  Urbanacity: Not reported | *n* = 693  Age: *M*_age_ = 12.94±0.54  Gender: 48.1% female  Demographics: Not reported | *n* = 480  Age: 12.94±0.54  Gender: 41% female  Demographics: Not reported | Title: Activity and Motivation in Physical EDucation  Duration: 14–15 months  Description: The intervention is a multicomponent school-based PA program designed to enhance students’ performance in mathematics. |
| Lynch et al., 2015 [31] | To use a collaborative approach between a medical center, the private sector and local school staff to assess the feasibility of using the Zamzee Program in the school-based setting to improve PA levels in children. | Observational study (Pre-post comparison) | United States  Urbanacity: Not reported | *n* = 11  Age: 8-14  Gender:  73% female  Demographics: Not reported | N/A | Title: Zamzee Program  Duration: 7 weeks  Description**:** The Zamzee Program is a wearable activity meter linked to a motivational website designed for children aged 8–14 years. In this study, the program was implemented in an elementary school setting to track and promote PA among participants over a 7-week period. The device recorded the minutes of MVPA and the associated website provided a platform for children and their caregivers to review activity results, complete PA challenges, and earn rewards. The study aimed to assess the feasibility and usability of the program in a school-based setting. |
| Martin & Fairclough, 2008 [32] | To examine whether an extra-curricular teaching intervention could increase MVPA in girls during dance lessons without compromising lesson objectives. | N-RCT: Quasi-experimental design | United Kingdom  Urbanacity: Not reported | *n* = 15  Age: 11-12  Gender: 100% female  Demographics: Not reported | N/A | Title: Extra-Curricular Dance Teaching Intervention  Duration: Not reported  Description: The intervention involved an extra-curricular dance program aimed at increasing MVPA among secondary school girls during dance lessons. Specific details regarding the structure and content of the intervention were not provided in the available summary. The study focused on assessing whether the intervention could enhance MVPA levels without compromising the educational objectives of the dance lessons. |
| Mateo-Orcajada et al., 2023a [33] | To examine changes in adolescents’ health following a compulsory 10-week after-school intervention using mobile step-tracking applications. The study also aimed to compare the benefits of different mobile applications used in the intervention. | Longitudinal study | Spain  Urbanacity: Not reported | *n* = 240  Age: 12-16  Gender: 190 females and 210 males  Demographics: Not reported | *n* = 140  Age: 12-16  Gender: not reported  Demographics: Not reported | Title: Mobile Step-Tracking Application Intervention  Duration: 10 weeks  Description: Participants were required to use assigned mobile step-tracking applications—such as Pokémon Go, Pacer, Strava, and MapMyWalk—at least three times per week outside of school hours. Each session mandated a minimum of 5,000 steps (approximately 3.19 km). Participants who did not meet this requirement were excluded from the study. The intervention aimed to assess the impact of these applications on adolescents’ PA levels and health outcomes. |
| Mateo-Orcajada et al., 2023b [34] | To examine the differences in the effectiveness of a mobile application-based intervention on PA levels, body composition, and physical fitness in adolescents, with a focus on gender and academic year variations. | RCT | Spain  Urbanacity: Not reported | *n* = 240  Age: 12-16  Gender: 190 females and 210 males  Demographics: Not reported | *n* = 140  Age: 12-16  Gender: Not reported  Demographics: Not rpoeted | Title: Mobile App-Based PA Promotion Program  Duration: 10 weeks  Description: Participants used a mobile application to promote PA, engaging with the app at least three times per week. The program featured a progressive step goal, starting at 7,000 steps per session in the first week and increasing to 12,520 steps per session by the tenth week. The intervention aimed to help adolescents achieve recommended levels of MVPA. |
| McKenzie et al., 2014 [35] | To examine the design, development, and feasibility of a mobile active videogame (M-AVG), “Pirate Adventure”, aimed at promoting PA and fundamental movement skills among primary school-aged children in an afterschool setting. | N-RCT: Quasi-experimental design | Australia  Urbanacity: Not reported | *n* = 14  Age: 5-10 (*M*_age_ = 6.8±1.97)  Gender: 5 females and 9 males  Demographics: Not reported | N/A | Title: Pirate Adventure Mobile Active Videogame (M-AVG)  Duration: Approximately 11 minutes per session  Description: Pirate Adventure is a mobile active videogame designed to promote PA and fundamental movement skills among primary school-aged children in an afterschool setting. The game involves children using mobile phones to scan Quick Response codes, solve clues, and complete PA and movement challenges. |
| Mischenko et al., 2021 [36] | To experimentally demonstrate the potential of the “Footbag” pedagogical game technology to increase children's motivation for motor activity. | N-RCT: Quasi-experimental design | Russia  Urbanacity: Not reported | *n* = 20  Age: 11-12  Gender: Not reported  Demographics: Not reported | *n* = 20  Age: 11-12  Gender: Not reported  Demographics: Not reported | Title: “Footbag-freestyle” Game Program  Duration: 30 lessons over the academic year, constituting 28.6% of total PE time  Description: The experimental group participated in “Footbag-freestyle” lessons integrated into their PE curriculum. These lessons focused on learning and practicing various Footbag techniques, such as catching the ball with the foot, rebounding, and different types of stalls. The control group continued with their regular PE classes without the Footbag intervention. |
| Mok et al., 2020 [37] | To examine the effectiveness of Brain Breaks PA Solutions in changing children’s attitudes toward PA. | RCT | Multinational: Croatia, Lithuania, Macedonia, Poland, Romania, Serbia, South Africa, and Turkey  Urbanacity: Not reported | *n* = 1914  Age: 8-11  Gender: 49.9% female  Demographics: Not reported | *n* = 1122  Age: 8-11  Gender: 52.0% female  Demographics: Not reported | Title: Brain Breaks PA Solutions  Duration: 4 months  Description: The intervention involved the integration of Brain Breaks videos into classroom sessions. These 3–5-minute videos provided movement-based activities designed to promote PA among students. The content included elements of health and nutrition education, social learning, environmental stewardship, core curricular learning, character development, and exposure to arts and culture, promoting a holistic approach to PA and overall development. The experimental group participated in these video sessions throughout the four-month intervention period. |
| Nation-Grainger, 2017 [38] | To examine the impact of wrist-worn digital PA monitors in providing individual feedback to adolescents, aiming to increase motivation and exercise levels in PE. | N-RCT: Quasi-experimental design | United Arab Emirates  Urbanacity: Not reported | *n* = 5  Age: 14-15  Gender: 100% male  Demographics: Majority are White | *n* = 5  Age: 14-15  Gender: 100% male  Demographics: Majority are White | Title: Wrist-Worn Digital PA Monitors  Duration: Not reported  Description: Participants in the intervention group wore wrist-worn digital PA monitors that provided individual feedback to increase awareness of exercise levels and progress. The intervention was designed to support competence, relatedness, and autonomy from Self-Determination Theory. It integrated elements of enjoyment and competition, similar to computer games, into PE lessons to enhance motivation. |
| Nigg et al., 2021 [39] | To examine the congruence between accelerometry and self-report methods for assessing MVPA and higher intensity PA in children. | Longitudinal study | United States  Urbanacity: Not reported | *n* = 564  Age: 9  Gender: 50% female  Demographics: 78% White, 14% Hispanic, 2% Black, 4% Other, 5% Two or more races | N/A | Title: Fuel for Fun Study  Duration: Evaluated over 12 months at three time points: baseline/pre-intervention in the fall of fourth grade, follow-up 1 post-intervention in the spring of fourth grade, and follow-up 2 in the fall of fifth grade.  Description: The Fuel for Fun Study is a multicomponent school- and family-based intervention aimed at promoting positive food and activity environments for fourth-grade students and their families. The program includes components such as cooking skills, nutrition education, and PA promotion, with the goal of encouraging healthier lifestyles among children. |
| Norris et al., 2018 [40] | To examine the effects of the “Virtual Traveller” intervention on PA, on-task behavior, and student engagement when delivered using classroom interactive whiteboards | RCT | United Kingdom  Urbanacity: Not reported | *n* = 113  Age: 8-9  Gender: 61 females and 52 males  Demogrphics: 53.1% White, 4.4% Mixed, 37.2% Asian or Asian British, 5.3% Black or Black British | *n* = 106  Age: 8-9  Gender: 47 females and 59 males  demographics: 42.5% White, 9.4% Mixed, 43.4% Asian or Asian British, 4.7% Black or Black British | Title: Virtual Traveller  Duration: 6-week intervention  Description: The Virtual Traveller intervention consisted of physically active lessons delivered using interactive whiteboards in classroom settings. These lessons, known as Virtual Field Trips, integrated educational content with related physical movements, aiming to increase PA, improve on-task behavior, and enhance student engagement. The program utilized existing classroom technology to create an immersive and active learning experience. |
| Oliver et al., 2006 [41] | To design and implement a 4 week elementary school curriculum unit, based around pedometer walking, and to quantify the PA levels of children prior to and during the unit implementation. | N-RCT: Quasi-experimental design | New Zealand  Urbanacity: Not reported | *n* = 78  Age: 8-10  gender: 52% female  Demographics: Not reported | N/A | Title: Integrated Pedometer-Based Curriculum Unit  Duration: 4 weeks  Description: A comprehensive curriculum unit integrating PA, pedometer use, and a  “virtual walk” around New Zealand across multiple subjects. The unit aimed to increase children’s PA levels while embedding learning objectives into the curriculum. Pedometers were used as motivational, educational, and measurement tools for PA. All school subjects (e.g., English, Mathematics, Science) in the 4-week unit were linked by a common theme of conducting a “virtual” walk around New Zealand. The study reported a significant increase in PA from baseline to posttest (approximately 2,000-4,000 steps/day) for participants classified as low-active at baseline. |
| Petrušič et al., 2022 [42] | To examine the effects of using digital placebo video games in the gym while teaching gymnastics elements on the amount and level of activity intensity of elementary school students. | RCT | Slovenia  Urbanacity: Not reported | *n* = 72  Age: 6-9 (*M*_age_ = 7.6±0.96)  Gender: 37 females and 35 mlales  Demographics: Not reported | *n* = 60  Age: 6-9 (*M*_age_ = 7.5±0.9)  Gender: 33 females and 27 males  Demographics: Not reported | Title: Digital Placebo Games in Gymnastics Education  Duration: Not reported  Description: The intervention group used digital placebo games created with laptops, projectors, and fake remotes to simulate a digital game experience during PE classes. Three artistic gymnastics elements were taught: forward roll, backward roll, and cartwheel. Accelerometer-based activity trackers measured activity time and intensity in both groups. |
| Remmers et al., 2020 [43] | To investigate changes of PA patterns in transition between primary and secondary school, and to add domain-specific insights of how, where, and when these changes occur. | Longitudinal study | Netherlands  Urbanacity: Not reported | *n* = 175  Age: *M*_age_ = 12.1  Gender: 86 females and 89 males  Demographics: Not reported | N/A | Title: Not reported  Duration: Data collected over 7 consecutive days during both the last year of primary school and the first year of secondary school.  Description: (No Intervention) Participants wore accelerometers and global positioning system (GPS) loggers for 7 consecutive days during their last year of primary school and again during their first year of secondary school. The collected data were integrated with Geographic Information System data to identify the contexts of PA, such as home, school, sports grounds, and transport. The study aimed to assess changes in PA patterns and the role of transport-related activity during the transition from primary to secondary school. |
| Robertson et al., 2018 [44] | To examine the effectiveness of the FitQuest exergame in increasing PA levels among children aged 10-11 years in primary schools. The study evaluated the impact of the game on step count, minutes spent in MVPA, and exercise self-efficacy. | RCT | United Kingdom  Urbanacity: Not reported | *n* = 111  Age: 10-11  Gender: Not reported  Demographics: Not reported | *n* = 104   Age: 10-11  Gender: Not reported  Demographics: Not reported | Title: FitQuest Exergame Intervention  Duration: Not reported  Description: The intervention involved the use of the FitQuest exergame system, which included features to support goal setting and social comparison through a leaderboard. Participants in the intervention group engaged with the FitQuest system during the study period. The system aimed to increase PA levels by encouraging children to achieve higher step counts and spend more time in MVPA. The control group continued with their usual activities without access to the FitQuest system. |
| Ruiter et al., 2022 [45] | To examine the effectiveness of a web-based Dutch parenting program designed to prevent overweight in children aged 9-13 years. | RCT | Netherlands  Urbanacity: Not reported | *n* = 252  Age: 9-13  Gender: Not reported  Demographics: Not reported | *n* = 223  Age: 9-13  Gender: Not reported  Demographics: Not reported | Title: Making a Healthy Deal with Your Child  Duration: 10 weeks  Description: A web-based parenting program aimed at teaching parents how to support their child’s healthy energy balance-related behaviors and handle daily situations that may jeopardize these behaviors. The program consisted of five 30-minute modules, each incorporating video fragments, a six-step problem-solving model, assignments, and feedback. Parents received emails after each module and reminder emails at weeks 7 and 12 to encourage completion. The control group received a standard brochure from the Dutch Nutrition Center regarding healthy eating and PA. |
| Schaefer et al., 2016 [46] | To examine the feasibility of fitness tracking among urban youth, particularly in low-resource communities, to understand how wearable technology can influence health and lifestyle behaviors. | Mixed methods design | United States  Urbnancity: Urban | *n* = 34  Age: 11-12  Gender: 12 females and 22 males  Demographics: 12 Mexican American, 6 African American, 4 mixed ethnicity, and 2 Asian American | N/A | Title: Project GETUP (Gaming to Educate Teens to Understand Personal Health)  Duration: 6 months  Description: Project GETUP is a multiyear collaborative research study integrating learning and health sciences with game design. Participants received Fitbit PA monitors to track their activity levels. Quantitative data were collected from Fitbit.com to assess engagement and activity patterns, while qualitative data were gathered through focus groups to explore motivators and barriers to Fitbit use. Participants also received training on using the Fitbit devices and syncing data to Fitbit.com. |
| Schofield et al., 2005 [47] | To examine the effectiveness of daily step count targets compared to time-based prescriptions for increasing health-related PA in low-active adolescent girls. | N-RCT: Quasi-experimental design | Australia  Urbanicity: Urban | *n* = 55  Age: *M*_age_ = 15.8±0.8  Gender: 100% female  Demographics: Primarily European descent | *n* = 24  Age: *M*_age_ = 15.8±0.8  Gender: 100% female  Demographics: Primarily European descent | Title: PA Self-Monitoring and Educative Program  Duration: 12 weeks  Description: The intervention involved a 12-week PA self-monitoring and educative program for low-active adolescent girls. Participants were divided into two intervention groups. The Pedometer Group set daily step count targets using pedometers to monitor their PA levels, while the Minutes Group set daily time-based goals for PA involvement without the use of pedometers. |
| Shayne et al., 2012 [48] | To examine the effects of exergaming on PA among third-grade students in a PE class. | N-RCT: Quasi-experimental design | United States  Urbanacity: Not reported | *n* = 4  Age: 3^rd^ grade  Gender: 100% male  Demographics: Not reported | N/A | Title: Exergaming in Physical Education  Duration: Not reported  Description: The intervention involved the use of exergaming equipment during physical education classes. Exergaming refers to video games that are also a form of exercise, encouraging players to be physically active while playing. The study compared the effects of exergaming and traditional PE on PA levels among the participants. |
| Stewart et al., 2004 [49] | To examine the effectiveness of the TAKE10! program in increasing student PA through academically linked activities within the public school system. | N-RCT: Quasi-experimental design | United States  Urbanacity: Not reported | *n* = 71  Age: 1^st^, 3^rd^, and 5^th^ grade  Gender: not reported  Demographics: African American (88%), Hispanic (7%), and Caucasian (5%) | N/A | Title: TAKE 10!  Duration: Not reported  Description: TAKE 10! is a classroom-based PA program designed to integrate 10-minute sessions of moderate to vigorous intensity activities with academic content. The program aims to increase student PA without requiring additional staff or extensive training, appealing to multiple learning styles. Activities are linked to academic subjects and implemented within the public school system. |
| Stoepker et al., 2018 [50] | To examine differences in PA accrual on the Walking Classroom (TWC) school days versus non-TWC school days. To explore on-task behavior before and after TWC. To better understand student and teacher experiences with the program. | Mixed methods design | United States  Urbanacity: Not reported | *n* = 22  Age: 9-11  Gender: 12 females and 10 males  Demographics: 12 Latino, 8 Caucasian, and 2 others | N/A | Title: The Walking Classroom  Duration: Not reported  Description: The Walking Classroom (TWC) is a program that integrates PA with academic content by providing structured classroom breaks where students engage in walking while listening to educational podcasts. The study aimed to assess differences in PA levels on days when TWC was implemented compared to non-TWC days, as well as to explore changes in on-task behavior and gather insights into student and teacher experiences with the program. |
| Sum & Leung, 2016 [51] | To examine the effectiveness of an Internet-based behavioral intervention for PA promotion among secondary school students. | Mixed methods design | Hong Kong, China  Urbanacity: Urban | *n* = 65  Age: 15-20  Gender: 35.4 % female  Demographics: Not reported | *n* = 62  Age: 15-20  Gender: 38.7 % female  Demographics: Not reported | Title: Let’s Exercise and be Active Daily (LEAD)  Duration: Not reported  Description: The LEAD program is an Internet-based behavioral intervention designed to promote PA among secondary school students. It includes a step-by-step exercise plan emphasizing the benefits of regular exercise, strategies to overcome barriers, and the importance of social support. The program aims to encourage students to engage in regular PA through structured online guidance and motivational content. |
| Szabo-Reed et al., 2020 [52] | To examine the effectiveness and sustainability of technology-delivered (PAAC-R) and classroom teacher-delivered (PAAC-T) activity breaks for increasing classroom MVPA in elementary school students. | RCT | United States  Urbanacity: Not reported | *n* = not specified (only number of schools reported = 16 schools)  Age: 2^nd^ and 3^rd^ graders progressing to grades 4-5  Gender: Not reported  Demographics: Not reported | N/A | Title: PAAC-R and PAAC-T  Duration: Not reported  Description: The study evaluated two types of activity breaks. PAAC-R (Remotely Delivered) involved activity breaks delivered through technology, such as videos or online platforms, aiming to increase MVPA among students. PAAC-T (Teacher-Delivered) consisted of activity breaks led by classroom teachers, incorporating PA into the classroom routine to promote MVPA. |
| Tománek et al., 2019 [53] | To examine effects of the technology-based PA intervention program on the attitudes of secondary school pupils towards PA. | N-RCT: Quasi-experimental design | Slovakia  Urbanacity: Not reported | *n* = 123  Age: 10-12  Gender: 58 females and 65 males  Demographics: Not reported | *n* = 106  age: 10-12 years  Gender: 56 females and 50 males  Demographics: Not reported | Title: Brain Breaks PA Program  Duration: 3 months  Description: The intervention group received 3-5 minute “Brain Breaks”—short PA sessions integrated into academic lessons using multimedia tools. These breaks aimed to improve students’ attitudes toward PA by providing brief, engaging movement opportunities during class. The control group continued with regular lessons without these activity breaks. |
| Vazou et al., 2021 [54] | To examine the feasibility and effectiveness of the Walkabouts program, a web-based physically active learning program, on attention and behavioral control of children from preschool to second grade. | N-RCT: Quasi-experimental design | United States  Urbanacity: Not reported | *n* = 158  Age: *M*_age_ = 6.01±1.45  Gender: 66 females and 92 males  Demographics: Caucasian 145 (91.8%), Asian 4 (2.5%), Hispanic 5 (3.2%), and other 3 (1.9%) | *n* = 87  Age: *M*_age_ = 5.33±1.44  Gender: 39 females and 48 males  Demographics: Predominantly Caucasian (87.5%), Asian (1.1%), and other (2.3%) | Title: Walkabouts  Duration: 7 weeks  Description: The Walkabouts program is a web-based physically active learning platform that integrates PA with academic content. It provides short, video-based activities designed for classroom implementation, aiming to enhance students’ attention and behavioral control by combining movement with learning tasks. Teachers can select activities that align with their curriculum, facilitating seamless integration of PA into daily lessons. |
| Vilardell-Dávila et al., 2023 [55] | To examine the impact of an educational intervention using digital media and face-to-face activities involving children, parents, and the school community on the level of PA and sedentary behavior among schoolchildren | N-RCT: Quasi-experimental design | Mexico  Urbanacity: Not reported | n = 201  Age: 6-10  Gender: Not reported  Demographics: Not reported | *n* = 167  Age: M_age_ = 8.1  Gender: Not reported  Demographics: Not reported | Title: Not reported  Duration: 12 months  Description: The intervention was a multi-component educational program aimed at reducing sedentary behavior and promoting PA among schoolchildren. It included digital media and face-to-face activities involving children, parents, and the school community. |
| West & Shores, 2014 [56] | To investigate how a technological intervention, HOPSports, impacts youth PA in PE classes. | Longitudinal pre-experimental single group design using  crossover treatment | United States  Urbanacity: Not reported | *n* = 387  Age: 4^th^-8^th^ grade (largest proportion in 6th grade: 34.9%)  Gender: 208 females and 179 males  demographics: Caucasian (63%), followed by African American (13.4%), Hispanic/Latino (14.7%), Multiracial (2.8%), and other (5.9%). | N/A | Title: HOPSports Technology System  Duration: Not reported  Description: The HOPSports technology system is designed to enhance PE classes by providing a multimedia approach to PA. The system includes a media cart and computer preloaded with over 250 lessons, which are projected onto a large screen. Students follow on-screen cues and instructions for various sport-specific and fitness activities, aiming to increase engagement and PA levels during PE sessions. |
| Ye et al., 2019 [57] | To examine the long-term effects of a school-based exergaming intervention on underserved minority children’s PA behaviors (MVPA, LPA, sedentary behavior) and cardiorespiratory fitness, hypothesizing that the exergaming intervention group will show greater improvements in these outcomes compared to the control group. | N-RCT: Quasi-experimental design | United States  Urbanacity: Not reported | *n* = 36  Age: *M*_age_ = 9.23±0.62  Gender: 16 females and 20 males  Demographics: African American 30 (83.3%), Non-hispanic American 3 (8.3%), and others 3 (8.4%) | *n* = 45  Age: *M*_age_ = 9.23±0.62   \|  \| \| --- \|   Gender: 13 females and 22 males  demographics: African American 14 (31.3%), Non-hispanic American 22 (48.9%), and others 9 (20.0%) | Title: School-Based Exergaming Intervention  Duration: 8 months  Description: The intervention consisted of setting up nine exergaming stations in a large classroom within the intervention school. Each station was equipped with an Xbox 360 or Nintendo Wii, featuring games such as Just Dance, Wii Fit, Gold’s Gym Cardio Workout, and Kinect Sports. The setup allowed two children to play simultaneously, with rotations every 10 minutes to maintain engagement and interest. The intervention school’s children participated in a once-weekly 50-minute exergaming session during recess throughout the school year, while the control school continued regular recess. PA levels were measured using ActiGraph GT3X+ accelerometers, and cardiorespiratory fitness was assessed via the half-mile run. Measurements were taken at baseline, mid-intervention (four months), and post-intervention (eight months). |
| Zhu & Dragon, 2016 [58] | To examine student interest and PA in technology-integrated PE lessons to determine whether their situational interest and PA levels changed over time within these lessons | N-RCT: Quasi-experimental design | United States  Urbanacity: Not reported | *n* = 27  Age: *M*_age_ = 11.04±0.33  Gender: 20 females and 7 males  Demographics Asian: 7.41%, African American: 3.70%, Latino: 37.04%, and Caucasian: 51.85% | n = 26  Age: Not reported  Gender: 18 females and 8 males  Demographics Asian: 7.69%, African American: 15.38%, Latino: 0%, Caucasian: 73.09%, and other: 3.84% | Title: Technology-Integrated PE Lessons  Duration: Not reported  Description: The intervention group participated in PE lessons that incorporated mobile technology to enhance PA and situational interest. Tools such as quick response (QR) codes and iPad applications were utilized to engage students. PA levels were measured using ActiGraph accelerometers, and the Situational Interest Scale was employed to assess student interest. The control group participated in the same PE lessons without the integration of technology. |

*Note:* PA: physical activity; PE: physical education; LPA: light physical activity; MVPA:moderate to vigorous physical activity; RCT: randomized controlled trials; N-RCT: non-randomized controlled trials

**Table S2**

*Technology-related information*

| **Source** | **Type of Technology Used** | **Context in Which Technology Used** | **Role of Technology for the Intervention** |
| --- | --- | --- | --- |
| Balasekaran et al., 2021 [1] | Video resources (Brain Breaks®) | In the classroom | Intervention subject |
| Botagariyev et al., 2024 [2] | Accelerometers (ActiGraph GT3X+), social media platform (Faecebook) | During physical education, expanding to home/online | Communication tool, measurement tool |
| Brackney et al., 2021 [3] | Activity trackers (Vivosmart HR+31) | Before school | Measurement tool |
| Buchele Harris & Chen, 2018 [4] | Activity trackers (Fitbits) | In the classroom | Intervention subject, measurement tool |
| Caillaud et al., 2022 [5] | Activity trackers (Misfit Ray©), mobile app (iEngage) | During school | Educational tool, intervention subject, measurement tool |
| Chen et al., 2020 [6] | Accelerometers (ActiGraph GT3X+) | During physical education | Measurement tool |
| Coknaz et al., 2019 [7] | Active video game | In the classroom | Intervention subject |
| Cook et al., 2014 [8] | Web-based platform (The HELENA Activ-O-Meter) | In the classroom | Communication tool, intervention subject |
| Coombes & Jones, 2016 [9] | Accelerometers (ActiGraph GT1M), smartcards and sensors (Beat Boxes) | Before school, expanding to home/online | Intervention subject, measurement tool |
| Corepal et al., 2019 [10] | Accelerometers (ActiGraph GT3X+), pedometers (Fitbit Zip), mobile app (Fitbit mobile app), web-based platform (The StepSmart Challenge website) | During school, in the classroom, expanding to home/online | Communication tool, intervention subject, measurement tool |
| Costigan et al., 2018 [11] | Accelerometers (GENEActiv) | During physical education, during school | Measurement tool |
| Crossley et al., 2019 [12] | 3D Printer, accelerometers (model or brand not specified) | During physical education | Intervention subject, measurement tool |
| Duck et al., 2021 [13] | Accelerometers (ActiGraph GT3X+), web-based platform (UNICEF Kid Power Bands) | In the classroom, expanding to home/online | Intervention subject, measurement tool |
| Duncan et al., 2019 [14] | Pedometers (Sealed NL-1000), web-based platform | In the classroom, expanding to home/online | Management tool, measurement tool |
| Erwin et al., 2013 [15] | Accelerometers (Computer Science and Applications [CSA] Actigraph) | In the classroom | Intervention subject, measurement tool |
| Evans et al., 2017 [16] | Activity trackers (Fitbit Zip and Fitbit Charge) | In the classroom | Intervention subject, measurement tool |
| Fogel et al., 2010 [17] | Exergaming equipment (Kanomi Dance Dance Revolution [DDR], Gamercize with Sony Play Station Batman and Robin, Three Rivers Game Cycle with Sony Play Station Monster 4 X 4, Electronic Sports Dog Fighter Simulator, Cateye Virtual Bike with Sony Play Station, Nintendo Wii Sports Baseball, Nintendo Wii Sports Tennis, Nintendo Wii Boxing, iTech Fitness XrBoard, Fit Interactive 3 Kick), Personal digital assistants (**These devices were used to collect duration data on the dependent variables). | During physical education | Intervention subject, measurement tool |
| Galy et al., 2019 [18] | Activity trackers (Misfit and GENEActiv trackers), mobile app (Bepatient [digital education app]) | In the classroom | Educational tool, intervention subject |
| González-Cutre et al., 2014 [19] | Video resources (types not specified) | During physical education | Intervention subject |
| Ha et al., 2022 [20] | Accelerometers (ActiGraph wGT3X-BT), Fun to Move@JC Sport Band, Kinect Infrared Camera (Microsoft Kinect), Raspberry Pi Devices (for data transfer gateways) | During physical education, in the classroom | Intervention subject, measurement tool |
| Hands et al., 2011 [21] | Pedometers (Yamax Digiwalker SW200) | In the classroom, expanding to home/online | Measurement tool |
| Hartwig et al., 2019 [22] | Accelerometers (ActiGraph wGT3X), pedometers (Yamax Digiwalker SW200 | During physical education | Measurement tool |
| Huang et al., 2019 [23] | A Web-based platform (designed with a self-management strategy and supplemented with a geographical information system (GIS) mapping function) | In the classroom, expanding to home/online | Communication tool, intervention subject |
| Jung et al., 2021 [24] | Accelerometers (ActiGraph wGT3X) | In the classroom | Educational tool, intervention subject, measurement tool |
| Koorts et al., 2020 [25] | Activity trackers (Fitbit Flex), mobile app (Fitbit), social media (Facebook) | During school | Communication tool, intervention subject, measurement tool |
| Layne et al., 2022 [26] | Accelerometers (model or brand not specified), heart rate monitors (model or brand not specified) | During physical education | Intervention subject, measurement tool |
| Lee et al., 2012 [27] | Pedometers (Omron HJ-720ITC) | During physical education; during school, in the classroom, expanding to home/online | Measurement tool |
| Lee & Gao, 2020 [28] | Accelerometers (ActiGraph GT3X+), iPads, mobile apps (Garage Band, Educreation Coach’s Eye, Scoreboard, Team Shake Interval Timer, Stopwatch) | During physical education | Educational tool, intervention subject, measurement tool |
| Lubans et al., 2014 [29] | Mobile app (ATLAS), web-based platform, pedometers (model or brand not specified) | During physical education, during school | Communication tool, intervention subject, measurement tool |
| Lubans et al., 2018 [30] | Accelerometers (ActiGraph GT3X+) | During physical education | Measurement tool |
| Lynch et al., 2015 [31] | Activity trackers (The Zamzee meter), web-based platform | In the classroom | Intervention subject, measurement tool |
| Martin & Fairclough, 2008 [32] | Accelerometers (ActiGraph GT3X+) | After school | Measurement tool |
| Mateo-Orcajada et al., 2023a [33] | Mobile apps (Strava, Pacer, MapMyWalk, and Pokémon Go) | After school, expanding to home/online | Intervention subject, measurement tool |
| Mateo-Orcajada et al., 2023b [34] | Mobile apps (Strava, Pacer, MapMyWalk, and Pokémon Go) | During physical education | Intervention subject |
| McKenzie et al., 2014 [35] | Computer software (Android Development Kit), smartphones (Google Nexus 4); QR code | After school | Educational tool, intervention subject, measurement tool |
| Mischenko et al., 2021 [36] | Active video game (Footbag technology-based exercise game program) | During physical education | Intervention subject |
| Mok et al., 2020 [37] | Video resources (HOPSports Brain Breaks®) | In the classroom | Intervention subject |
| Nation-Grainger, 2017 [38] | Smartwatches (Samsung Gear Smart Watch) | In physical education | Intervention subject, measurement tool |
| Nigg et al., 2021 [39] | Accelerometers (GENEActive) | In the classroom, expanding to home/online | Measurement tool |
| Norris et al., 2018 [40] | Accelerometers (ActiGraph GT1M), interactive whiteboards | In the classroom | Educational tool, intervention subject, measurement tool |
| Oliver et al., 2006 [41] | Pedometers (Yamax SW-200 DIGI Walker) | During physical education, in the classroom | Measurement tool |
| Petrušič et al., 2022 [42] | Accelerometers (MMOXX1.07), digital Placebo games | During physical education | Intervention subject, measurement tool |
| Remmers et al., 2020 [43] | Accelerometers (ActiGraph GT3X+), Global Positioning System (GPS) | Not specified (described as school setting) | Measurement tool |
| Robertson et al., 2018 [44] | Accelerometers (NL 1000 piezoelectric), smartphones | During physical education, during school | Intervention subject, measurement tool |
| Ruiter et al., 2022 [45] | Web-based platform (e-learning program) | Not specified (described as school setting) | Educational tool |
| Schaefer et al., 2016 [46] | Activity trackers (Fitbit) | After school | Intervention subject, measurement tool |
| Schofield et al., 2005 [47] | Pedometers (model or brand not specified) | During school | Intervention subject, measurement tool |
| Shayne et al., 2012 [48] | Exergaming equipment (Konomi Dance, Monster 434 DDR, Nintendo Wii [baseball, tennis, boxing], and Game Bikes), Personal digital Assistants (PDAs) | During physical education | Intervention subject |
| Stewart et al., 2004 [49] | Accelerometers, video resources (TAKE 10!) | In the classroom | Intervention subject, measurement tool |
| Stoepker et al., 2018 [50] | Audio player, pedometers (FitStepPro) | In the classroom | Intervention subject, measurement tool |
| Sum & Leung, 2016 [51] | Web-based platform | During physical education | Intervention subject |
| Szabo-Reed et al., 2020 [52] | Accelerometers (ActiGraph GT3X+), activity timer | In the classroom | Intervention subject, measurement tool |
| Tománek et al., 2019 [53] | Video Animations | In the classroom | Educational tool, intervention subject |
| Vazou et al., 2021 [54] | Web-based platform | In the classroom | Educational tool |
| Vilardell-Dávila et al., 2023 [55] | Text messages, web-based platform | In the classroom, expanding to home/online | Communication tool, educational tool |
| West & Shores, 2014 [56] | Accelerometers (Actigraph GT1M), web-based platform (HOPSports system [a media cart and computer system]) | During physical education | Intervention subject, measurement tool |
| Ye et al., 2019 [57] | Accelerometers (ActiGraph GT3X+), Exergaming equipment (Xbox 360 and Nintendo Wii) | During school | Intervention subject, measurement tool |
| Zhu & Dragon, 2016 [58] | Accelerometers (ActiGraph GT3X+),  Instant Heart Rate monitor (Azumio 2015 ver.), iPads, web-based platform (Edmodo educational technology platform), QR codes | During physical education | Intervention subject, management tool, measurement tool |

**Table S3**

*Effectiveness of Technology-infused PA Interventions*

| **Source** | **PA-Related Variable Measured** | **Data Collection Instrument** | **Statistical Results (e.g., p-value, effect size, percentage increase/decrease)** | **Key findings** |
| --- | --- | --- | --- | --- |
| Balasekaran et al., 2021 [1] | Attitude towards PA benefits | Physical Activity Scale (PAS) | Effect sizes ranged from 0.08 to 0.37. All PA scale scores increased significantly more in the experimental group compared to the control group. | The Brain Breaks® intervention positively influenced students' attitudes towards PA. |
| Botagariyev et al., 2024 [2] | PA level | ActiGraph GT3X+ Accelerometers | A statistically significant increase in PA across all schools, with an average increase of 1053 counts per minute. | The introduction of digital technologies in PE classes significantly increased students' PA levels. |
| Brackney et al., 2021 [3] | PA level | Physical Activity Questionnaire-Child (PAQ-C); Vivosmart HR+31 activity trackers | The intervention group averaged 18 more minutes of daily MVPA than the comparison group. A multivariate analysis confirmed significant differences in weekly PA intensity between the groups (*p* < .05). | The before-school, play-based dance program was effective in promoting PA among second-grade students. |
| Buchele Harris & Chen, 2018 [4] | PA level | Fitbit activity tracker | The PAEB-C group showed significantly higher steps and minutes of very active and fairly active minutes compared to the Fitbit-O group. Statistical results: *p* = 0.014 for steps; *p* = 0.021 for very active minutes; *p* = 0.002 for fairly active minutes. | The technology-enhanced PA intervention effectively improved real-time PA among students. |
| Caillaud et al., 2022 [5] | PA level | Misfit Ray© activity trackers; GENEActiv accelerometers; Physical Activity Questionnaire for Older Children (PAQ-C) | A 30% increase in daily steps (*p* < .001). MVPA increased by 5 minutes per day in the week following the program (*p* = .023). Participants met or exceeded daily step goals on 55% of days. | The iEngage program successfully helped adolescents achieve PA goals, with boys increasing their goals and achievements faster than girls. |
| Chen et al., 2020 [6] | PA knowledge | ActiGraph GT3X+ Accelerometers | The treatment group improved from 53.0% to 61.5% in PA knowledge, outperforming the control group, which decreased from 58.2% to 51.4%. | The HIIT-based curriculum was effective in improving PA knowledge among middle school students. |
| Coknaz et al., 2019 [7] | PA enjoyment; Self-perception about PA | Children and Youth Physical Self Perception Profile (CY-PSPP); Physical Activity Enjoyment Scale (PACES-SF) | No significant gender differences in PA enjoyment scales for Active Video Games. However, Active Video Games notably contributed to children's PA. | Active Video Games positively impacted children's physical self-perception, aiding in PA promotion and obesity prevention. |
| Cook et al., 2014 [8] | PA level; Psychosocial determinants of PA | International Physical Activity Questionnaire adapted for adolescents (IPAQ-A); Self-report questionnaires | Significant increases in MVPA and MPA in leisure time (*p* = 0.002 for both). Significant increases in VPA in leisure time (*p* = 0.025) and at school (*p* = 0.039). | Interventions promoting PA in adolescents should address environmental barriers at school to improve PA outcomes. |
| Coombes & Jones, 2016 [9] | PA level | ActiGraph GT1M accelerometers | No significant difference in total PA between the intervention and control schools (p = 0.823). However, the intervention school had a significantly smaller decline in MVPA minutes (*p* = 0.020). | The intervention had a positive association between Beat Box engagement and increased MVPA during school commute times. |
| Corepal et al., 2019 [10] | PA level; Social support for PA | ActiGraph accelerometers; Fitbit Zip pedometers | The StepSmart Challenge was found to be feasible and acceptable for promoting PA among adolescents. | The StepSmart Challenge intervention was an acceptable and effective approach to promoting PA in adolescents. |
| Costigan et al., 2018 [11] | PA level | GENEActiv accelerometers | Group-by-time interaction effects for MPA and VPA were small and moderate. Adjusted difference for VPA: 1.70 minutes per day (95% *CI*: -1.96 to 5.36, *p* = 0.354). | Embedding HIIT within the school day moderately increased VPA among adolescents, though its overall impact on total PA was modest. |
| Crossley et al., 2019 [12] | PA level | Accelerometers | 59% of youths reported increased awareness of their PA levels after receiving 3D models, though the effect diminished slightly by the final model. | 3D models enhanced youth awareness of PA levels and had potential as a motivational tool for PA promotion. |
| Duck et al., 2021 [13] | PA level | Actigraph GT3X accelerometers | No significant difference in PA levels between the control and intervention groups (control: *p* = 0.05, intervention: *p* = 0.12). Both groups showed decreased PA from baseline to post-intervention. | The wearable activity tracker intervention did not improve PA levels, as both groups showed a decrease in PA. |
| Duncan et al., 2019 [14] | PA level | Sealed NL-1000 pedometers | Significant increases in weekday PA at home (*p* < 0.001) and weekend PA (*p* < 0.001). Greatest improvements in PA observed in children from socioeconomically deprived schools. | The Healthy Homework intervention led to significant increases in children's PA, particularly in socioeconomically deprived schools. |
| Erwin et al., 2013 [15] | PA level | Computer Science and Applications (CSA) Actigraph | 67.92% of indoor recess time was spent in PA, with 22.22% in MVPA. Boys accumulated a higher percentage of MVPA and total PA compared to girls. No interaction between grade level and sex for MVPA (*p* = .44). | PA dance videos during indoor recess effectively increased children's PA levels compared to sedentary indoor activities. |
| Evans et al., 2017 [16] | PA level | Fitbit Charge monitor | No significant differences in MVPA and steps per day between groups at baseline and follow-up (*p* > 0.05). | Although wearable PA monitors were feasible, they did not significantly increase PA levels, possibly due to lack of individualized goals and adult-directed opportunities. |
| Fogel et al., 2010 [17] | PA level | Survey | Exergaming resulted in higher PA levels compared to standard PE. | Exergaming is a promising intervention for increasing PA among inactive children in PE classes, though further research is needed to assess its long-term effects. |
| Galy et al., 2019 [18] | PA level | Misfit activity trackers | Daily steps increased by 15%, and adherence to PA recommendations increased by 27%, especially among the least active adolescents. | Technology-supported educational programs with self-monitoring via activity trackers successfully changed adolescents' PA behaviors, particularly in remote areas. |
| González-Cutre et al., 2014 [19] | Motivation towards PA in leisure time; Attitude, subjective norm, behavioral control, and intention towards PA**;** Habitual PA | Questionnaires | Significant differences in the experimental group for several variables (*p* < 0.05). | The intervention led to increased motivation and PA levels, emphasizing the importance of autonomy support from teachers, peers, and parents in promoting PA. |
| Ha et al., 2022 [20] | PA level | ActiGraph wGT3X-BT accelerometers | Strong correlation (*r* = 0.795) between the developed system and accelerometer-measured PA. The Sport Band captured data on 78% of days, compared to 55% for accelerometers. | The Fun to Move@JC Sport Band has shown strong validity and can effectively measure children's PA. |
| Hands et al., 2011 [21] | PA levels; Parental reports of child PA | Yamax Digiwalker SW200 pedometer; Parent questionnaire; Student survey | The experimental group exhibited a 16.1% higher mean VPA than the control group, equating to an additional 3.85 minutes of activity per day (*p* = 0.04). The intervention did not significantly affect MPA. | Children should be given opportunities for self-determination by being encouraged to make choices about their PA. |
| Hartwig et al., 2019 [22] | PA level | ActiGraph GT3X+ accelerometers; Yamax Digi-Walker Step Pedometers | The mean discrepancy between the monitoring system and ActiGraph accelerometers in MVPA was 1.6%, with lessons averaging 24.2% MVPA. A correlation coefficient of 0.896 was found between step counts and accelerometer-determined %MVPA. | The PA monitoring system effectively provided valid feedback on the proportion of PE lesson time spent in MVPA. It presents opportunities to increase PA in classrooms or group-based settings. |
| Huang et al., 2019 [23] | PA level | A modified Chinese version of the Child/Adolescent Activity Log | The self-management group showed significantly higher PA levels than the knowledge-only and control groups (*p* < .001). The self-management group also demonstrated a better understanding of personal PA habits. | The web-based intervention, integrating self-management and GIS mapping, effectively increased PA levels among school children. |
| Jung et al., 2021 [24] | PA level; Motivation and engagement in PA; The alignment of PA with educational goals and the use of technology in teaching | ActiGraph GT3X+ accelerometers; RFID badge system; Fitness Education Index | Self-reported PA levels gradually improved as students received feedback from objective data and teacher instruction. Significant differences were observed in self-reported PA between 4th and 5th graders. | The community-based participatory approach used in Project SMART was time-consuming but yielded numerous benefits and unintended outcomes. |
| Koorts et al., 2020 [25] | Perceived changes in motivation, awareness, and encouragement for PA | Survey; Fitbit Flex and mobile app | Adolescents reported that the Fitbit Flex increased their motivation (70.8%) and awareness (78.2%) of PA. | The RAW-PA intervention was well-received by adolescents and teachers, but suffered from low engagement and adherence. While initial positive effects on motivation and awareness were observed, they were unlikely to be sustained. |
| Layne et al., 2022 [26] | PA level | Accelerometers | The Sport Education model group engaged in significantly more MVPA during lessons than the traditional instruction group (61.01% vs 51.74% of class time, p < 0.001). No significant gender differences in MVPA were observed. | The Sport Education model, enhanced with heart rate monitors, significantly increased 4th graders' MVPA compared to traditional instruction. |
| Lee et al., 2012 [27] | PA level | Omron HJ-720ITC pocket pedometers | The intervention group showed a significantly greater increase in aerobic steps (467 more steps) compared to the control group. | The intervention, combining self-efficacy theory and pedometers, effectively increased PA among adolescent girls, as evidenced by the increase in aerobic steps. |
| Lee & Gao, 2020 [28] | PA level; PA-related psychosocial beliefs: Self-efficacy, outcome expectancy, social support, and enjoyment | ActiGraph GTX3+ accelerometers; Questionnaires | Significant differences were observed in both LPA and MVPA between the experimental and control groups. | The integration of mobile applications in PE classes significantly impacted children's PA levels, improving both LPA and MVPA. |
| Lubans et al., 2014 [29] | PA level | Pedometers; ATLAS mobile app | Nearly half of participants found the push prompt messages helpful, but only 44% agreed the app itself was enjoyable, compared to 95% who enjoyed the overall ATLAS program. | Participants reported moderate satisfaction with the app but were more positive about the overall intervention. Post-program, participants showed strong intentions to engage in regular PA. |
| Lubans et al., 2018 [30] | PA level | ActiGraph GT3X+ accelerometers | The intervention had a significant effect on the proportion of PE lesson time spent in MVPA (*p* < 0.001). | The AMPED intervention significantly increased MVPA during PE lessons, indicating its effectiveness in promoting PA during PE. |
| Lynch et al., 2015 [31] | PA level | Self-Administered Physical Activity Checklist (SAPAC) survey; Zamzee Activity Meter | No significant changes in PA levels over the 7-week period (median increase of 17 minutes, not statistically significant). Activity levels were higher on school days (40 minutes) than on non-school days (15 minutes). | The Zamzee program was usable but did not significantly improve PA levels. Larger, longer-duration studies are needed to evaluate effectiveness. |
| Martin & Fairclough, 2008 [32] | PA level | ActiGraph GT3X+ accelerometers | The intervention increased girls' MVPA during lessons from 29.7% to 34.9% (*p* < 0.05) over eight weeks. | Deliberate planning and modified teaching approaches effectively increased PA levels in dance lessons, supporting dance as a significant contributor to PA for girls. |
| Mateo-Orcajada et al., 2023a [33] | PA level | Physical Activity Questionnaire for Adolescents (PAQ-A) | Significant increase in PA in the experimental group compared to the control group (*p* = 0.039). | The mandatory use of mobile step-tracking apps after school hours led to improvements in PA levels. |
| Mateo-Orcajada et al., 2023b [34] | PA level | Physical Activity Questionnaire for Adolescents (PAQ-A) | Females used mobile apps more than males (71.1% vs 50.0%, *p* < 0.001). Older adolescents (4th academic year) used the apps more than younger adolescents (74.4% vs 53.8%, *p* < 0.001). | Females and older adolescents were more likely to use mobile apps to promote PA than males and younger adolescents. |
| McKenzie et al., 2014 [35] | PA enjoyment | Gameplay statistic data; A modified version of the Physical Activity Enjoyment Scale (PACES) | The "Pirate Adventure" game design successfully created an enjoyable treasure hunt, with children averaging 11 minutes of activity per game. Most participants (9 out of 13) expressed a desire to play again. | The game design allowed PA to be incorporated flexibly and was adaptable based on the participant group and available context. |
| Mischenko et al., 2021 [36] | PA motivation | Questionnaires | The experimental group showed a universal increase in motivation for PA among all boys, while only some boys in the control group experienced this increase. Boys in the experimental group achieved daily MVPA durations exceeding 60 minutes, outperforming the control group by 56.6%. | The "Footbag" game program significantly increased PA motivation in 11-12-year-old boys compared to a control group. |
| Mok et al., 2020 [37] | Attitude towards PA: Self-efficacy, learning, benefits, importance, personal best, fun, and fitness | Questionnaires | The Brain Breaks PA Solutions intervention led to significant improvements in student attitudes toward PA. | The Brain Breaks intervention effectively integrated PA into the classroom with minimal disruption. |
| Nation-Grainger, 2017 [38] | PA level | Samsung Gear Smart Watches | No significant changes in calories burned or distance covered at the start and end of the intervention. However, there was a significant increase in calories burned and distance covered from the second to the sixth week of the intervention. | Digital feedback on exercise levels significantly boosted students' motivation, thereby enhancing their PA levels during PE classes. |
| Nigg et al., 2021 [39] | PA level | GENEActive accelerometers; Questionnaires | Self-reported PA exceeded accelerometer-measured VPA by 9-15 minutes daily, but was lower for MPA, LPA, and MVPA. The practical discrepancy in MVPA was minimal (5.5 minutes per day). | Questionnaires and accelerometers aligned well in measuring MVPA among upper elementary students but captured distinct data for LPA. Multiple methods should be used to assess PA levels comprehensively. |
| Norris et al., 2018 [40] | PA level | Actigraph GT1M accelerometers; Children’s Activity Rating Scale (CARS); Observing Teachers and Pupils in Classrooms (OPTIC); Student Engagement Instrument—Elementary version (SEI-E) | The experimental group showed higher school-day MVPA during the first intervention period (*p* < 0.05). They also demonstrated significantly higher weekend-day LPA at the 1-week follow-up (*p* < 0.05). | The Virtual Traveller intervention did not increase overall PA levels during school or on weekends but did increase PA during active lessons, with no negative impact on student engagement. |
| Oliver et al., 2006 [41] | PA level | Yamax SW-200 DIGI Walker pedometers | No significant change in PA for the entire sample when comparing baseline to intervention weekdays. Over half of participants achieved over 15,000 steps daily. PA levels were significantly higher on weekdays compared to weekends. | The integrated curriculum approach using pedometers to increase PA in children is feasible. |
| Petrušič et al., 2022 [42] | PA level | MMOXX1.07 accelerometers | The intervention group exhibited significantly higher levels of LPA and MVPA than the control group across all three gymnastics elements (*p* < 0.001). No significant differences in VPA were observed between the two groups. | Incorporating digital placebo games into PE classes effectively increased LPA and MVPA during gymnastics instruction in elementary schools. |
| Remmers et al., 2020 [43] | PA level; Context-specific PA patterns | ActiGraph GT3X+ accelerometers; Global Positioning System (GPS) logger with Personal Activity and Location Measurement System (PALMS) | Boys engaged in more MVPA during and after school than girls. A significant decrease in MVPA was noted from primary to secondary school, with less pronounced declines during school hours. | Children spent more time outside of school grounds in secondary school, leading to declines in PA intensity during school hours. |
| Robertson et al., 2018 [44] | PA level; Self-efficacy for PA | NL 1000 piezoelectric accelerometers; Questionnaires | The FitQuest intervention did not significantly affect self-efficacy for PA or MVPA levels compared to the control group. | The FitQuest exergame was not successful in increasing self-efficacy or MVPA among 10-11-year-olds. |
| Ruiter et al., 2022 [45] | PA behaviors: Time spent playing outside, participation in organized sports | Questionnaires | No statistically significant differences in PA behaviors between the intervention and control groups, even after adjusting for multiple tests. | The web-based parenting program was well-received by parents but did not have significant beneficial effects on children's PA behaviors compared to the control group. |
| Schaefer et al., 2016 [46] | PA level | Fitbit activity tracker | Minor increase in engagement over time (r = 0.368), though not statistically significant. Significant reduction in daily device usage by students within the first month. | Wearable devices alone do not significantly alter PA practices among urban youth. Comprehensive support, including appropriate technology, training, curriculum, incentives, and safe PA opportunities, is crucial. |
| Schofield et al., 2005 [47] | PA level | 3-Day Physical Activity Recall (3DPAR); Pedometers | The pedometer intervention group showed a significantly greater increase in step count compared to the control group at post-intervention (*p* = 0.03, *ES* = 0.13). At mid-intervention, the increase was more pronounced in the pedometer group (*p* = 0.04, *ES* = 0.10). | Pedometers and daily step count targets resulted in enhanced PA outcomes in the short term (6 weeks). Both pedometer and MIN groups showed similar improvements after 12 weeks. |
| Shayne et al., 2012 [48] | PA level | Personal digital Assistants (PDAs) | Exergaming stations like Monster 434 and DDR led to higher levels of PA, whereas Wii Sports resulted in lower levels. Students engaged in PA for 82.5% of available time during exergaming, compared to 48.8% in traditional PE. | Exergaming is a promising intervention for increasing PA in elementary school children. |
| Stewart et al., 2004 [49] | PA level | Accelerometers | MVPA exhibited average energy intensities ranging from 4.98 to 7.19 across the first, third, and fifth grades, with no significant differences observed between these grade levels. | The TAKE 10! program was effective in promoting meaningful PA in the classroom across all grade levels. |
| Stoepker et al., 2018 [50] | PA level | FitStepPro pedometers (Gopher Sport) | No significant difference in PA accrual between TWC and non-TWC days. | The TWC program did not increase PA accrual compared to non-walking classroom days. |
| Sum & Leung, 2016 [51] | PA level | International Physical Activity Questionnaire (IPAQ) | The internet-based PA promotion program did not significantly increase PA levels or stages of change in the intervention group compared to the control group. | The internet-based program was not effective in increasing PA levels in secondary school students. |
| Szabo-Reed et al., 2020 [52] | PA level; PA enjoyment; Self-efficacy for PA | ActiGraph GT3X+ accelerometers | The PAAC trials confirmed the feasibility of incorporating MVPA in classroom settings. Classroom-based MVPA contributed significantly to achieving daily MVPA levels and enhancing total daily MVPA in elementary school children. | Classroom-based MVPA can effectively contribute to meeting recommended daily MVPA levels, improving overall PA among elementary school children. |
| Tománek et al., 2019 [53] | Attitude towards PA | A standardized questionnaire provided by the Ministry of Education of the Slovak Republic. | The experimental group showed a significant increase in attitude scores compared to the control group (*p* = 0.007, g = 0.51). | The "Brain Breaks" classroom-based PA program positively impacted secondary school students’ attitudes toward PA. |
| Vazou et al., 2021 [54] | PA level | System for Observing Student Movement in Academic Routines and Transitions (SOSMART) | The Walkabouts intervention benefitted kindergarten students more than other grade levels. | The Walkabouts program was perceived as feasible, physically active, and enjoyable by teachers, especially for kindergarten students. |
| Vilardell-Dávila et al., 2023 [55] | PA level | Questionnaires adapted from validated Activity and Inactivity of Mexican Students (CAINM) questionnaire | The intervention did not significantly affect the time spent in MVPA. | The intervention had no impact on students’ MVPA levels. |
| West & Shores, 2014 [56] | PA level | Actigraph GT1M accelerometers | The HOPS technology intervention yielded significantly higher MVPA levels compared to traditional PE. Girls showed more MVPA during dance, and boys during sports. Older students (grades 7-8) showed greater MVPA gains, although younger students were more active overall. | The HOPS intervention effectively promoted MVPA across genders in PE classes, with activity choices being key to achieving higher activity levels. |
| Ye et al., 2019 [57] | PA level | ActiGraph GT3X+ accelerometers | The intervention group exhibited a significant increase in MVPA over time compared to the control group (*p* < 0.05). A significant time-by-group interaction was also observed for LPA (*p* < 0.01). | The school-based exergaming intervention was effective in increasing MVPA among children, but the control group showed greater improvements in LPA. |
| Zhu & Dragon, 2016 [58] | PA level | ActiGraph GT3X+ accelerometers | Students in technology-integrated PE lessons had lower PA levels compared to the control group. | Mobile technology-integrated PE may not be effective in increasing students' PA levels over a short duration. |

*Note:* PA: physical activity; PE: physical education; LPA: light physical activity; MPA: moderate physical activity; VPA: vigorous physical activity; MVPA:moderate to vigorous physical activity

**Appendix A**

*PRISMA 2020 Checklist*

| **Section and Topic** | **Item #** | **Checklist item** | **Location where item is reported** |
| --- | --- | --- | --- |
| **TITLE** | | |  |
| Title | 1 | Identify the report as a systematic review. | Page 1 |
| **ABSTRACT** | | |  |
| Abstract | 2 | See the PRISMA 2020 for Abstracts checklist. | Pages 2-3 |
| **INTRODUCTION** | | |  |
| Rationale | 3 | Describe the rationale for the review in the context of existing knowledge. | Pages 5-6 |
| Objectives | 4 | Provide an explicit statement of the objective(s) or question(s) the review addresses. | Page 6 |
| **METHODS** | | |  |
| Eligibility criteria | 5 | Specify the inclusion and exclusion criteria for the review and how studies were grouped for the syntheses. | Page 8 |
| Information sources | 6 | Specify all databases, registers, websites, organisations, reference lists and other sources searched or consulted to identify studies. Specify the date when each source was last searched or consulted. | Page 7 |
| Search strategy | 7 | Present the full search strategies for all databases, registers and websites, including any filters and limits used. | Page 7, Table 1 |
| Selection process | 8 | Specify the methods used to decide whether a study met the inclusion criteria of the review, including how many reviewers screened each record and each report retrieved, whether they worked independently, and if applicable, details of automation tools used in the process. | Page 9 |
| Data collection process | 9 | Specify the methods used to collect data from reports, including how many reviewers collected data from each report, whether they worked independently, any processes for obtaining or confirming data from study investigators, and if applicable, details of automation tools used in the process. | Pages 9-10 |
| Data items | 10a | List and define all outcomes for which data were sought. Specify whether all results that were compatible with each outcome domain in each study were sought (e.g. for all measures, time points, analyses), and if not, the methods used to decide which results to collect. | Pages 9-10 |
|  | 10b | List and define all other variables for which data were sought (e.g. participant and intervention characteristics, funding sources). Describe any assumptions made about any missing or unclear information. | Pages 9-10 |
| Study risk of bias assessment | 11 | Specify the methods used to assess risk of bias in the included studies, including details of the tool(s) used, how many reviewers assessed each study and whether they worked independently, and if applicable, details of automation tools used in the process. | Pages 10-11 |
| Effect measures | 12 | Specify for each outcome the effect measure(s) (e.g. risk ratio, mean difference) used in the synthesis or presentation of results. | Page 10 |
| Synthesis methods | 13a | Describe the processes used to decide which studies were eligible for each synthesis (e.g. tabulating the study intervention characteristics and comparing against the planned groups for each synthesis (item #5)). | Page 10 |
|  | 13b | Describe any methods required to prepare the data for presentation or synthesis, such as handling of missing summary statistics, or data conversions. | N/A |
|  | 13c | Describe any methods used to tabulate or visually display results of individual studies and syntheses. | N/A |
|  | 13d | Describe any methods used to synthesize results and provide a rationale for the choice(s). If meta-analysis was performed, describe the model(s), method(s) to identify the presence and extent of statistical heterogeneity, and software package(s) used. | N/A |
|  | 13e | Describe any methods used to explore possible causes of heterogeneity among study results (e.g. subgroup analysis, meta-regression). | N/A |
|  | 13f | Describe any sensitivity analyses conducted to assess robustness of the synthesized results. | N/A |
| Reporting bias assessment | 14 | Describe any methods used to assess risk of bias due to missing results in a synthesis (arising from reporting biases). | N/A |
| Certainty assessment | 15 | Describe any methods used to assess certainty (or confidence) in the body of evidence for an outcome. | N/A |
| **RESULTS** | | |  |
| Study selection | 16a | Describe the results of the search and selection process, from the number of records identified in the search to the number of studies included in the review, ideally using a flow diagram. | Page 11 |
|  | 16b | Cite studies that might appear to meet the inclusion criteria, but which were excluded, and explain why they were excluded. | N/A |
| Study characteristics | 17 | Cite each included study and present its characteristics. | Page 13 |
| Risk of bias in studies | 18 | Present assessments of risk of bias for each included study. | Pages 18-19 |
| Results of individual studies | 19 | For all outcomes, present, for each study: (a) summary statistics for each group (where appropriate) and (b) an effect estimate and its precision (e.g. confidence/credible interval), ideally using structured tables or plots. | Supplementary Document Table S1 |
| Results of syntheses | 20a | For each synthesis, briefly summarise the characteristics and risk of bias among contributing studies. | Pages 14-18,  Supplementary Document  Table S1 |
|  | 20b | Present results of all statistical syntheses conducted. If meta-analysis was done, present for each the summary estimate and its precision (e.g. confidence/credible interval) and measures of statistical heterogeneity. If comparing groups, describe the direction of the effect. | N/A |
|  | 20c | Present results of all investigations of possible causes of heterogeneity among study results. | N/A |
|  | 20d | Present results of all sensitivity analyses conducted to assess the robustness of the synthesized results. | N/A |
| Reporting biases | 21 | Present assessments of risk of bias due to missing results (arising from reporting biases) for each synthesis assessed. | N/A |
| Certainty of evidence | 22 | Present assessments of certainty (or confidence) in the body of evidence for each outcome assessed. | N/A |
| **DISCUSSION** | | |  |
| Discussion | 23a | Provide a general interpretation of the results in the context of other evidence. | Pages 19-25 |
|  | 23b | Discuss any limitations of the evidence included in the review. | Pages 25-26 |
|  | 23c | Discuss any limitations of the review processes used. | Page 25 |
|  | 23d | Discuss implications of the results for practice, policy, and future research. | Pages 26-27 |
| **OTHER INFORMATION** | | |  |
| Registration and protocol | 24a | Provide registration information for the review, including register name and registration number, or state that the review was not registered. | Page 7 |
|  | 24b | Indicate where the review protocol can be accessed, or state that a protocol was not prepared. | N/A |
|  | 24c | Describe and explain any amendments to information provided at registration or in the protocol. | N/A |
| Support | 25 | Describe sources of financial or non-financial support for the review, and the role of the funders or sponsors in the review. | Page 27 |
| Competing interests | 26 | Declare any competing interests of review authors. | Page 28 |
| Availability of data, code and other materials | 27 | Report which of the following are publicly available and where they can be found: template data collection forms; data extracted from included studies; data used for all analyses; analytic code; any other materials used in the review. | Page 28 |

**Figure S1**

*Results of the quality assessment: RoB 2*


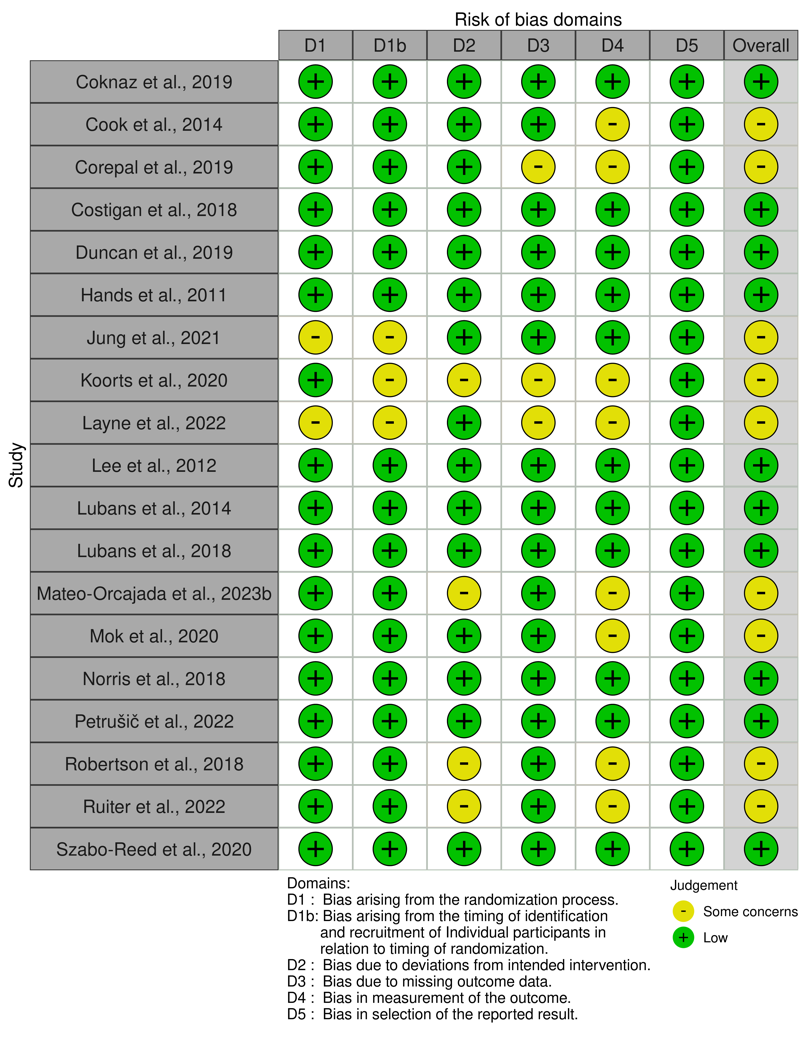


**Figure S2**

*Results of the quality assessment: ROBINS-I*


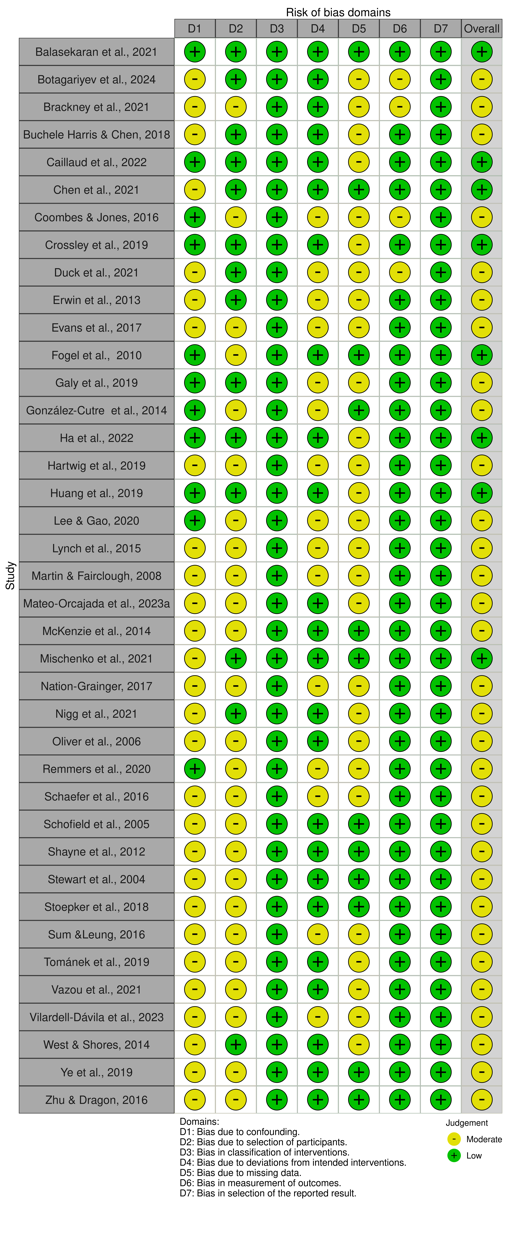


**References**

1. Balasekaran G, Ibrahim AAB, Cheo NY, Wang PK, Kuan G, Popeska B, et al. Using Brain-Breaks® as a technology tool to increase attitude towards physical activity among students in Singapore. Brain Sci. 2021;11(6):784.

2. Botagariyev T, Mambetov N, Aralbayev A, Mukhanbetaliyev A, Ispulova R, Akhmetov N. Web-Based Technologies in Middle School Physical Education. Retos: nuevas tendencias en educación física, deporte y recreación. 2024(51):172-8.

3. Brackney D, Daughtridge E, Jensen M, Childers S, Tocci N, Wilson T, et al. Feasibility of play-based dance to promote physical activity in second grade students. Am J Health Educ. 2021;52(5):266-75.

4. Buchele Harris H, Chen W. Technology-enhanced classroom activity breaks impacting children’s physical activity and fitness. J Clin Med. 2018;7(7):165.

5. Caillaud C, Ledger S, Diaz C, Clerc G, Galy O, Yacef K. iEngage: A digital health education program designed to enhance physical activity in young adolescents. PloS one. 2022;17(10):e0274644.

6. Chen S, Liu Y, Androzzi J, Wang B, Gu X. High-intensity interval training-based fitness education in middle school physical education: a limited-efficacy study. J Teach Phys Educ. 2020;40(4):566-76.

7. Coknaz D, Mirzeoglu AD, Atasoy HI, Alkoy S, Coknaz H, Goral K. A digital movement in the world of inactive children: favourable outcomes of playing active video games in a pilot randomized trial. Eur J Pediatr. 2019;178:1567-76.

8. Cook TL, De Bourdeaudhuij I, Maes L, Haerens L, Grammatikaki E, Widhalm K, et al. Psychosocial determinants and perceived environmental barriers as mediators of the effectiveness of a web-based tailored intervention promoting physical activity in adolescents: The HELENA Activ-O-Meter. J Phys Act Health. 2014;11(4):741-51.

9. Coombes E, Jones A. Gamification of active travel to school: A pilot evaluation of the Beat the Street physical activity intervention. Health Place. 2016;39:62-9.

10. Corepal R, Best P, O’Neill R, Kee F, Badham J, Dunne L, et al. A feasibility study of ‘The StepSmart Challenge’to promote physical activity in adolescents. Pilot Feasibility Stud. 2019;5:1-15.

11. Costigan SA, Ridgers ND, Eather N, Plotnikoff RC, Harris N, Lubans DR. Exploring the impact of high intensity interval training on adolescents’ objectively measured physical activity: Findings from a randomized controlled trial. J Sports Sci. 2018;36(10):1087-94.

12. Crossley SGM, McNarry MA, Eslambolchilar P, Knowles Z, Mackintosh KA. The tangibility of personalized 3D-printed feedback may enhance youths’ physical activity awareness, goal setting, and motivation: Intervention study. J Med Internet Res. 2019;21(6):e12067.

13. Duck AA, Hall KC, Klamm M, Temple M, Robinson JC. Physical activity and fitness: the feasibility and preliminary effectiveness of wearable activity tracker technology incorporating altruistic motivation in youth. J Spec Pediatr Nurs. 2021;26(1):e12313.

14. Duncan S, Stewart T, McPhee J, Borotkanics R, Prendergast K, Zinn C, et al. Efficacy of a compulsory homework programme for increasing physical activity and improving nutrition in children: a cluster randomised controlled trial. Int J Behav Nutr Phys Act. 2019;16:1-12.

15. Erwin H, Koufoudakis R, Beighle A. Children's physical activity levels during indoor recess dance videos. J Sch Health. 2013;83(5):322-7.

16. Evans EW, Abrantes AM, Chen E, Jelalian E. Using novel technology within a school‐based setting to increase physical activity: a pilot study in school‐age children from a low‐income, Urban Community. Biomed Res Int. 2017;2017(1):4271483.

17. Fogel VA, Miltenberger RG, Graves R, Koehler S. The effects of exergaming on physical activity among inactive children in a physical education classroom. J Appl Behav Anal. 2010;43(4):591-600.

18. Galy O, Yacef K, Caillaud C. Improving pacific adolescents’ physical activity toward international recommendations: Exploratory study of a digital education app coupled with activity trackers. JMIR Mhealth Uhealth. 2019;7(12):e14854.

19. González-Cutre D, Ferriz R, Beltrán-Carrillo VJ, Andrés-Fabra JA, Montero-Carretero C, Cervelló E, et al. Promotion of autonomy for participation in physical activity: A study based on the trans-contextual model of motivation. Educ Psychol. 2014;34(3):367-84.

20. Ha AS, Cheng J, Chan CH, Jiang G, Yang Y, Ng JY. Examining the criterion validity of two scalable, information technology-based systems designed to measure the quantity and quality of movement behaviours of children from Hong Kong primary schools: a cross-sectional validation study. BMJ open. 2022;12(8):e060448.

21. Hands B, Larkin D, Rose E, Parker H, Smith A. Can young children make active choices? Outcomes of a feasibility trial in seven‐year‐old children. Early Child Dev Care. 2011;181(5):625-37.

22. Hartwig TB, del Pozo‐Cruz B, White RL, Sanders T, Kirwan M, Parker PD, et al. A monitoring system to provide feedback on student physical activity during physical education lessons. Scand J Med Sci Sports. 2019;29(9):1305-12.

23. Huang S-j, Hung W-c, Shyu M-L, Chang K-c, Chen C-K. Web-based intervention to promote physical activity in Taiwanese children. J Pediatr Nurs. 2019;45:e35-e43.

24. Jung Y, Burson SL, Julien C, Bray DF, Castelli DM. Development of a school-based physical activity intervention using an integrated approach: project SMART. Front Psychol. 2021;12:648625.

25. Koorts H, Salmon J, Timperio A, Ball K, Macfarlane S, Lai SK, et al. Translatability of a wearable technology intervention to increase adolescent physical activity: mixed methods implementation evaluation. J Med Internet Res. 2020;22(8):e13573.

26. Layne T, Simonton K, Irwin C. Effects of a sport education instructional model and heart rate monitor system on the physical activity and jump rope performance of fourth grade students. J Phys Educ Sport. 2022;22(4):889-99.

27. Lee LL, Kuo YC, Fanaw D, Perng SJ, Juang IF. The effect of an intervention combining self‐efficacy theory and pedometers on promoting physical activity among adolescents. J Clin Nurs. 2012;21(7‐8):914-22.

28. Lee JE, Gao Z. Effects of the iPad and mobile application-integrated physical education on children’s physical activity and psychosocial beliefs. Phys Educ Sport Peda. 2020;25(6):567-84.

29. Lubans DR, Smith JJ, Skinner G, Morgan PJ. Development and implementation of a smartphone application to promote physical activity and reduce screen-time in adolescent boys. Front Public Health. 2014;2:42.

30. Lubans DR, Beauchamp MR, Diallo TM, Peralta LR, Bennie A, White RL, et al. School physical activity intervention effect on adolescents' performance in mathematics. Med Sci Sports Exerc. 2018;50(12):2442-50.

31. Lynch B, Jones A, Biggs B, Kaufman T, Cristiani V, Kumar S, et al. Implementing child-focused activity meter utilization into the elementary school classroom setting using a collaborative community-based approach. J Community Med Health Educ. 2015;5(6):379.

32. Martin S, Fairclough S. Dance as a health enhancing physical activity: An extra-curricular teaching intervention to increase physical activity in girls dance. afPE PM. 2008;3(1):37-41.

33. Mateo-Orcajada A, Abenza-Cano L, Albaladejo-Saura MD, Vaquero-Cristóbal R. Mandatory after-school use of step tracker apps improves physical activity, body composition and fitness of adolescents. Educ Inf Technol. 2023;28(8):10235-66.

34. Mateo-Orcajada A, Vaquero-Cristóbal R, Abenza-Cano L. Gender and academic year as moderators of the efficacy of mobile app interventions to promote physical activity in adolescents: a randomized controlled trial. Humanit Soc Sci Commun. 2023;10(1):1-15.

35. McKenzie S, Bangay S, Barnett LM, Ridgers ND, Salmon J. Design elements and feasibility of an organized multiplayer mobile active videogame for primary school-aged children. Games Health J. 2014;3(6):379-87.

36. Mischenko Ny, Kolokoltsev M, Romanova E, Vorozheikin A, Ivanova E, Suslina I. Schoolchildren's motivation to increase motor activity using the footbag freestyle game. J Phys Educ Sport. 2021;21(5):2657-63.

37. Mok MMC, Chin M-K, Korcz A, Popeska B, Edginton CR, Uzunoz FS, et al. Brain breaks® physical activity solutions in the classroom and on attitudes toward physical activity: a randomized controlled trial among primary students from eight countries. Int J Environ Res Public Health. 2020;17(5):1666.

38. Nation-Grainger S. ‘It’s just PE’till ‘It felt like a computer game’: using technology to improve motivation in physical education. Res Papers in Educ. 2017;32(4):463-80.

39. Nigg CR, Burg X, Lohse B, Cunningham-Sabo L. Accelerometry and self-report are congruent for children’s moderate-to-vigorous and higher intensity physical activity. J Meas Phys Behav. 2021;4(2):187-94.

40. Norris E, Dunsmuir S, Duke-Williams O, Stamatakis E, Shelton N. Physically active lessons improve lesson activity and on-task behavior: A cluster-randomized controlled trial of the “Virtual Traveller” Intervention. Health Educ Behav. 2018;45(6):945-56.

41. Oliver M, Schofield G, McEvoy E. An integrated curriculum approach to increasing habitual physical activity in children: a feasibility study. J Sch Health. 2006;76(2):74-9.

42. Petrušič T, Bogataj Š, Štemberger V. Teaching gymnastics elements in elementary school with placebo-effect digital games: cluster-randomised controlled trial. Kinesiol Slov. 2022;28(3): 5-15.

43. Remmers T, Van Kann D, Kremers S, Ettema D, De Vries SI, Vos S, et al. Investigating longitudinal context-specific physical activity patterns in transition from primary to secondary school using accelerometers, GPS, and GIS. Int J Behav Nutr Phys Act. 2020;17:1-14.

44. Robertson J, Macvean A, Fawkner S, Baker G, Jepson RG. Savouring our mistakes: Learning from the FitQuest project. Int J Child Comput Interact. 2018;16:55-67.

45. Ruiter EL, Molleman GR, Kleinjan M, Kraiss JT, Ten Klooster PM, van der Velden K, et al. The effectiveness of a web-based Dutch parenting program to prevent overweight in children 9–13 years of age: Results of a two-armed cluster randomized controlled trial. PloS one. 2022;17(10):e0276168.

46. Schaefer SE, Ching CC, Breen H, German JB. Wearing, thinking, and moving: testing the feasibility of fitness tracking with urban youth. Am J Health Educ. 2016;47(1):8-16.

47. Schofield L, Mummery WK, Schofield G. Effects of a controlled pedometer-intervention trial for low-active adolescent girls. Med Sci Sports Exerc. 2005;37(8):1414-20.

48. Shayne RK, Fogel VA, Miltenberger RG, Koehler S. The effects of exergaming on physical activity in a third-grade physical education class. J Appl Behav Anal. 2012;45(1):211-5.

49. Stewart JA, Dennison DA, Kohl HW, Doyle JA. Exercise level and energy expenditure in the TAKE 10! in-class physical activity program. J Sch Health. 2004;74(10):397-400.

50. Stoepker P, Dauenhauer B, McCall T. Effects of a classroom walking program on physical activity accrual and on-task behavior. Phys Educ. 2018;75(3):498-514.

51. Sum RKW, Leung EFL. Efficacy of using Internet-based interventions for physical activity promotion in a Hong Kong Secondary School: An action research approach. Cogent Educ. 2016;3(1):1221026.

52. Szabo-Reed AN, Washburn RA, Greene JL, Ptomey LT, Gorczyca A, Lee RH, et al. Physical activity across the curriculum (PAAC3): Testing the application of technology delivered classroom physical activity breaks. Contemp Clin Trials. 2020;90:105952.

53. Tománek Ľ, Cihová I, Luptáková G, Antala B, Chin M-k, Šagát P. Effect of technology based programme "Brain Breaks" on the pupils' attitudes towards physical activity in secondary schools. Pertanika J Soc Sci & Hum. 2019;27(S3):47-60.

54. Vazou S, Long K, Lakes KD, Whalen NL, et al. “Walkabouts” integrated physical activities from preschool to second grade: feasibility and effect on classroom engagement. Child Youth Care For; 2021; 50:39-55.

55. Vilardell-Dávila A, Martínez-Andrade G, Klünder-Klünder M, Miranda-Lora AL, Mendoza E, Flores-Huerta S, et al. A multi-component educational intervention for addressing levels of physical activity and sedentary behaviors of schoolchildren. Int J Environ Res Public Health. 2023;20(4):3003.

56. West ST, Shores KA. Does HOPSports promote youth physical activity in physical education classes? Phys Educ. 2014;71(1):16-40.

57. Ye S, Pope ZC, Lee JE, Gao Z. Effects of school-based exergaming on urban children’s physical activity and cardiorespiratory fitness: A quasi-experimental study. Int J Environ Res Public Health. 2019;16(21):4080.

58. Zhu X, Dragon LA. Physical activity and situational interest in mobile technology integrated physical education: A preliminary study. Acta Gymn. 2016;46(2):59-67.
